# Antioxidant Defenses and Poly(ADP-Ribose) Polymerase (PARP) Activity Provide “Radioresilience” Against Ionizing Radiation-Induced Stress in Dwarf Bean Plants

**DOI:** 10.3390/antiox14030261

**Published:** 2025-02-25

**Authors:** Bruno Hay Mele, Anna Rita Bianchi, Valeria Guerretti, Mariagabriella Pugliese, Anna De Maio, Carmen Arena

**Affiliations:** 1 Department of Biology, University of Naples Federico II, Via Cinthia, 4, 80126 Naples, Italy; 2 Department of Physics “Ettore Pancini”, University of Naples Federico II, Via Cinthia, 4, 80126 Naples, Italy

**Keywords:** oxidative stress, photosynthetic activity, polyphenols, resilience capability, ROS, X-rays

## Abstract

Exposure to ionizing radiation (IR) poses a significant risk for all organisms. Although plants are generally more resistant than animals, radiation still impacts their structure and function. Plant resistance to ionizing radiation is a pivotal property to guarantee their survival. This study evaluates bean leaves’ early and long-term responses to oxidative stress induced by ionizing radiation. To assess the early response, we measured a battery of photosynthetic efficiency and oxidative stress markers after exposure of dwarf bean plants to X-ray doses of 0.3, 10, 50, and 100 Gy. We observed that doses started to impact photosynthetic activity at 50 Gy and that markers aggregate in two kinds of behaviors. To test the capacity to recover from radiation-induced damages, 50 Gy-irradiated plants were evaluated with the same markers 3-, 10-, 12-, and 20-days post-irradiation. Dwarf beans displayed remarkable resilience, recovering photosynthetic activity to pre-stress level after three days and pigment content after ten days. The remodulation of oxidative stress markers is slower and more complex, with catalase and total polyphenols failing to recover completely and residual antioxidant activity after twenty days. Despite that, PARP activity recovers to pre-irradiation after three days. The restoration of photosynthesis to pre-irradiated conditions highlights the DNA-repairing efficiency of poly(ADP-ribose) polymerase and antioxidant machinery in providing resilience to radiation-induced oxidative stress. Understanding resilience mechanisms sheds light on the ability of plants to survive and thrive in radiation-intense environments, such as space or radioactively contaminated areas.

## 1. Introduction

As sessile organisms, plants are exposed to environmental fluctuations and sudden, unfavorable stresses. Consequently, they evolved a remarkable physiological plasticity that allows them to persist in diverse environments and withstand various degrees of harshness [1]. Among the myriad environmental stressors, pollution poses a significant threat to plant health, with radioactive contamination standing out as particularly detrimental [2]. Sources of radioactive pollution include improper disposal of nuclear waste and catastrophic nuclear accidents, both of which release harmful levels of ionizing radiation into the environment [3]. Like all biological systems, plants exposed to radioactive materials may be severely affected by direct energy transfer and indirectly by reactive oxygen species (ROS), which include ions, atoms, and various molecules and radicals, such as hydrogen peroxide (H_2_O_2_), singlet oxygen (^1^O_2_), the superoxide anion radical (O_2_•−), and the hydroxyl radical (OH•). Among these, the hydroxyl radical poses the greatest threat to tissues due to its high reactivity, which enables it to oxidize numerous cellular components, including lipids, proteins, carbohydrates, and DNA. This oxidation process leads to lipid peroxidation, resulting in the formation of reactive lipid derivatives that can inflict oxidative damage on other biomolecules [4].

Exposure to IR has various effects, including temporary tissue damage, mutagenesis, and impaired growth and reproduction.

Research conducted after major nuclear disasters, such as those in Chernobyl, Ukraine (1986) and Fukushima, Japan (2011) has significantly informed our understanding of plant responses to radioactive environments [5,6]. Research following the Chernobyl incident revealed that exposure to sparsely and densely ionizing radiation altered population dynamics and structure, disrupted DNA and protein functions, and induced genetic mutations in exposed organisms. For example, population-level changes included reduced seed germination rates and altered species compositions in contaminated zones.

Laboratory and field studies have shown that ionizing radiation exerts diverse effects on plant metabolism, growth, and reproduction, with outcomes mediated by several factors. These include the plant’s developmental stage at exposure time, physiological and morphological traits, and genetic makeup [7,8].

The impact of ionizing radiation also depends on the dose and type of radiation, categorized by linear energy transfer (LET). In some cases, radioactive contamination has been linked to embryo lethality, dwarfism, and altered floral structures [6].

While plant response regarding damage is usually proportional to dose, low doses may occasionally induce stimulatory responses (hormesis) [9]. This effect has been observed in some irradiated crops, with low doses promoting taller growth, higher yields, and improved drought resilience [10,11].

Radiation-induced responses are driven by direct, non-selective, and potentially transient damage to molecular structures and by stochastic DNA damage. The latter can disrupt regulative processes, cause infertility, and induce mutations, including deletions, base substitutions, and chromosomal rearrangements [12]. Genetic studies have shown that exposure to heavy-ion beams induces mutations at a much higher frequency than exposure to X-rays due to differences in their higher LET that cause targeted, more severe damage [13]. Additionally, it has been shown that low doses of heavy-ion radiation can produce non-lethal mutations, thus broadening the spectrum of genetic variability in irradiated plants [14].

At the functional level, numerous studies have investigated the effects of ionizing radiation on the photosynthetic apparatus, revealing impacts at various stages of the photosynthetic process from light-harvesting reactions to the pathway of carbon fixation [15,16,17]. Photosystem II (PSII) is highly sensitive to radiation due to its highly oxidative microenvironment [18], with damage often targeting the D1 protein, a critical component of the photosynthetic electron transport chain. This vulnerability can disrupt electron flow, reducing photosynthetic efficiency and limiting energy production for growth, repair, and reproduction, ultimately reducing fitness. Photosynthetic pigments are also affected, as evidenced in bean plants exposed to X-ray doses of 50 and 100 Gy (mimicking extreme acute exposure conditions, like a nuclear disaster). These plants exhibited a significant reduction in chlorophyll and carotenoid content and impaired Rubisco activity [19]. This reduction led to photobleaching and diminished light-harvesting capacity. These findings highlight the detrimental effects of radiation on photosynthetic efficiency.

Plants often display a remarkable ability to tolerate radiation, i.e., radioresistance. This trait is attributed to integrated genetic, anatomical, and physiological adaptations. Collectively, these characteristics enable plants to withstand the challenges posed by radioactive contamination more effectively than animals [7,20].

For example, following the nuclear disasters at Chernobyl and Fukushima, plants in highly contaminated areas demonstrated significant resilience to both acute and chronic radiation doses, a phenomenon linked to a variety of molecular and biochemical mechanisms [6].

At the molecular level, exposure to ionizing radiation often triggers the hyperactivation of poly(ADP-ribose) polymerases (PARPs) [19], a class of enzymes crucial for identifying damaged DNA and facilitating its repair [21]. Specifically, PARP-1 and PARP-2 play essential roles in detecting and signaling DNA strand breaks due to ionizing radiation, thereby initiating the repair process through the ADP-ribosylation of chromatin components [22]. These DNA repair mechanisms are energy intensive, and a plant’s ability to activate protective strategies and repair damage depends heavily on its metabolic energy reserves.

PARPs may work synergistically with molecular defenses, such as antioxidant systems. Across these, phenolic compounds, such as phenols and flavonoids, play a critical role in scavenging radiation-generated ROS. These compounds act within mesophyll cells to mitigate oxidative stress and protect cellular structures from damage to enzymatic antioxidants, including superoxide dismutase (SOD) and catalase (CAT), further contributing to the neutralization of ROS [12,23]. Structural features of plant cells further enhance radioresistance. Thickened cell walls, protective cuticles, and trichomes (hairs) are among the traits that have been shown to help mitigate the damaging effects of ionizing radiation by diminishing its penetration through the cell [24].

PARP hyperactivation, antioxidant systems, and other molecular defenses often work synergistically within plant cells to combat radiation-induced stress. Species-specific traits and intrinsic characteristics influence which protective strategies are most effectively deployed, highlighting the complexity and variability of plant responses to ionizing radiation.

These adaptive mechanisms—spanning genetic, biochemical, and physiological domains—play a crucial role in determining plant resilience and recovery. The dynamic interplay between these strategies underscores the multifaceted nature of plant resilience and survival in radiation-exposed environments [2]. 

This study investigates the effect of X-ray exposure on the photosynthetic apparatus (F_v_/F_m_, chlorophyll content) and antioxidant defenses (polyphenols, antioxidant capacity, PARP, and CAT activity) of dwarf bean plants. We used non-parametric statistical tests to compare dose-dependent responses and applied robust nonlinear curve fitting to infer recovery dynamics over time. Additionally, we investigate the roles of antioxidant molecules, PARP activity, and enzymatic defenses from ROS in promoting plant resilience (the ability to converge toward pre-stress conditions after disturbance). To that aim, photosynthetic efficiency and biochemical marker levels were tracked over time to evaluate the plant’s ability to withstand and repair radiation-induced damage following exposure to a high X-ray dose (50 Gy). Understanding these mechanisms provides valuable insights for developing radiation-resilient crops and strategies for phytoremediation in contaminated areas but also for choosing species to be utilized for space research [25].

## 2. Materials and Methods

### 2.1. Plant Material and Growth Conditions

Seeds of dwarf bean (*Phaseolus vulgaris* L.) were purchased from Dom Sementi S.p.a. (Mortegliano, Udine, Italy) and sown in pots filled with peat/soil (1/1). Each pot was placed inside a growth chamber, equipped with an LED lighting system with a broad-spectrum white light at 35 cm from the light source and programmed at the following environmental conditions: a photosynthetic photon flux density (PPFD) of 200 ± 25 μmol photons m**^−^**^2^ s**^−^**^1^, a day/night temperature of 25/20 °C, a day/night relative humidity of 50/70%, and a 12/12 day/night photoperiod. All the plants were irrigated regularly to optimize their growth and development. When plants showed the first trifoliate leaf well expanded (vegetative stage), they were divided into two groups: one for the control and another for the irradiation procedure.

### 2.2. Irradiation Procedure

The X-rays were delivered by a Thomson tube (TR 300F, 250 kVp, dose rate 0.8 Gy × min**^−^**^1^, Stabilipan, Siemens) and filtered through a 1 mm thick copper foil. Physical dosimetry was conducted before each irradiation using an ionization chamber (Victoreen, Fluke Biomedical Division, WA, USA) to ensure reliable and consistent measures. Whole plants were irradiated every time, and the X-ray dose was measured, positioning the trifoliate leaf sample under the X-ray tube.

A 1 min irradiation was measured using a probe in rad; the values were subsequently converted to Gray (1 rad  =  0.01 Gy) at a specific distance. The dose in Gy was then used to estimate the total exposure time required to achieve the desired dose for each plant and treatment. Adjustments for plant self-shielding were not applied, as *P. vulgaris* at the vegetative stage has minimal foliar biomass.

To build dose–response curves, plants were irradiated at increasing doses of X-rays: 0.3, 10, 50, and 100 Gy. The dose–response curves aimed to find for *P. vulgaris* a threshold between putative positive outcomes at very low doses and negative consequences at high doses. To prevent the cumulative effects of ionizing radiation from confounding the specific response to each dose, five different plants were irradiated for each X-ray dose [16].

Additionally, five plants were maintained without irradiation and used as a control. A total of 25 plants and 25 leaves (one leaf per plant) were analyzed.

To test the plant’s capability to recover from high X-ray doses, a cohort of five bean plants exposed at a dose of 50 Gy was evaluated 30 min after irradiation (t = 0) and at 3, 10, 12, and 20 days from X-ray exposure. During the recovery period, irradiated plants were placed in the climatic chamber under the same environmental conditions experimented with before irradiation.

For all physiological measurements and biochemical essays, a sample size of five replicates (n = 5 leaves) from five different plants was used for each dose and recovery time.

### 2.3. Chlorophyll a Fluorescence Emission Measurements and Total Chlorophyll Determination

Chlorophyll a fluorescence measurement as well as photosynthetic pigment determination were carried out immediately (30 min) after irradiation at the described X-ray doses and through the recovery time points on fully expanded trifoliate leaves to assess plant retrieval capability.

Fluorescence emission was measured by a pulse amplitude-modulated fluorometer (Junior-PAM, Walz, Effeltrich, Germany). Measuring light of about 0.5 µmol photons m^−2^ s^−1^ was applied to leaves adapted to darkness for 30 min to obtain the basal fluorescence signal (F_0_). Then, after switching the frequency of measuring light at 20 kHz, a 1 s saturating pulse of 8000 photons µmol m^−2^ s^−1^ was applied to the leaf sample to record the maximum fluorescence (F_m_) and determine the maximum quantum efficiency of PSII primary photochemistry, calculated as F_v_/F_m_ = (F_m_ − F_0_)/F_m_ according to Krause e Weis [26]. The total chlorophyll content (a + b) was determined according to Lichtenthaler [27]. Fresh leaf samples were powdered with liquid nitrogen. Powdered material (0.020 g) was mechanically extracted in 100% acetone and centrifuged at 3000× *g* in a Labofuge GL (Heraeus Sepatech, Hanau, Germany) for 10 min. The sample absorbance was measured with a spectrophotometer (UV-VIS Cary 100, Agilent Technologies, Palo Alto, CA, USA) at wavelengths of 662 and 645 nm for chlorophyll *a* and chlorophyll *b*, respectively. Pigment concentration was expressed as mg g^−1^ of fresh weight (mg g^−1^ FW).

### 2.4. Nuclei Isolation and Poly (ADP-Ribose) Polymerase (PARP) Activity

The isolation of nuclei was performed according to [28]. All operations were carried out on ice or at 4 °C. Fresh leaves of dwarf beans were harvested, cut, and resuspended in 10 mM Tris-HCl (pH 7.0), 1 mM EDTA, 1 mM EGTA, 1 mM PhMeSO_2_F, 10 mM MgCl_2_, 5 mM β-mercaptoethanol, and 0.5% Triton X-100 (1/4, *w*/*v*) (buffer A). The samples were homogenized for 30–40 s at low speed by an Ultra Turrax T8 (IKA-WERKE). The homogenates were filtered and centrifuged at 1500× *g* for 30 min at 4 °C. The pellets containing nuclei were suspended in buffer A and were centrifuged as above three times. Finally, the pellets (nuclear fractions) were washed with buffer A without Triton X-100 (buffer B) and suspended in a small volume of buffer B containing 2% glycerol.

The ADP-ribosylating activity was determined as reported by Arena et al. [28]. In brief, the assay was carried out on isolated nuclei incubated in the presence of 0.4 mM [^32^P] NAD+ (10,000 cpm/nmole) in a 500 mM Tris-HCl buffer, pH 8.0; 50 mM MgCl_2_ and 10 mM DTT (reaction mixture). The reaction was stopped by adding ice-cold 20% trichloroacetic acid (*w*/*v*). Then, the mixture was filtered on Millipore filters (HAWPP0001, 0.45 μm; Millipore Sigma, Burlington, United States) and subjected to various washes using 7% trichloroacetic acid. The radioactivity was determined as the acid-insoluble material on a Beckman (model LS 1701; Beckman Coulter, Brea, United States) liquid scintillation spectrometer. The activity was measured as acid-insoluble radioactivity by liquid scintillation in a Beckman counter (model LS 1701; Beckman Coulter, Brea, United States).

### 2.5. Hydro- and Lipo-Soluble Antioxidant Capacity Measurement

Total hydro-soluble antioxidants were determined on fresh leaves following the procedure reported by Prieto et al. [29] on fresh leaves. The assay is based on the reduction in Mo(VI) to Mo(V) by the sample analyte and the following development of a green phosphate/Mo(V) complex at acidic pH. The samples (0.5 g) were frozen under liquid nitrogen and ground with a pestle and mortar to a fine powder. A volume of water (1 mL g^−1^) was added, and the suspension was homogenized and shaken for 1h at room temperature in the dark. The suspensions were centrifuged at 10,000 g for 15 min. The pellets were resuspended in another volume of solvent and centrifuged once more. The two supernatants were combined and kept at 4 °C. For the spectrophotometric determinations, 1 mL of reagent solution (0.6 M sulfuric acid, 28 mM sodium phosphate, and 4 mM ammonium molybdate) was added to aliquots of samples (0.1 mL) and incubated at 95 °C for 90 min. The absorbance of the aqueous solutions was measured at 695 nm by a spectrophotometer (uniSPEC 2 UV/VIS, Lab Logistics Group GmbH Labware, Meckenheim, Germany). The blank solution contained 1 mL of reagent solution, and the appropriate volume of the same solvent used for the sample was incubated under the same conditions as the rest of the samples. In the same experimental condition, the molar absorption coefficient ε (3400 ± 0.1 M^−1^ cm^−1^) value of the stock solution of ascorbic acid (AsA) was determined. Hydro-soluble antioxidant capacity was expressed as equivalents (μmol g^−1^ FW) of ascorbic acid (AsA).

Total lipo-soluble antioxidants were determined by the phosphomolybdenum method, combined with hexane extraction [29]. Hexane extracts (0.1 mL), containing lipo-soluble antioxidants, were mixed with 1 ml of reagent solution (0.6 M sulfuric acid, 28 mM sodium phosphate, and 4 mM ammonium molybdate) and incubated at 37 °C for 120 min with vigorous shaking.

The blank solution containing 1 mL of reagent solution and 0.1 mL of pure hexane was incubated as the samples. A stock solution of α-tocopherol (1 M) in hexane was used to determine the molar absorption coefficient ε (4000 ± 0.1 M^−1^ cm^−1^). Lipo-soluble antioxidant capacity was expressed as equivalent (nmol g^−1^ FW) of α-tocopherol.

### 2.6. Total Polyphenol Determination and Catalase Activity Assay

To quantify total polyphenol samples, fresh leaves (0.20 g) were extracted in methanol, kept overnight at 4 °C, and centrifuged at 11,000× *g* for 5 min.

The total polyphenol content was obtained according to Costanzo et al. [30]. Briefly, each extract was mixed with 10% Folin–Ciocâlteu reagent (1:1, *v*:*v*) and, after 3 min, with 700 mM Na_2_CO_3_ solution (1:5, *v*:*v*). Then, samples were incubated for 2 h in the dark at room temperature, and the absorbance was read by a spectrophotometer (Cary 100 UV-VIS, Agilent Technologies, Santa Clara, CA, USA) at 765 nm. The polyphenol concentration was expressed as mg of gallic acid equivalents per gram of dry weight (mg GAE g^−1^ DW) using a gallic acid calibration curve.

Catalase (CAT) activity (EC 1.11.1.6) was determined as reported by Arena et al. [28] on fresh leaf tissue. Briefly, CAT activity was measured on leaf extracts, following the protocol provided by the Catalase Assay Kit (Sigma-Aldrich, St. Louis, MO, USA), based on a colorimetric method in which the decomposition reaction of H_2_O_2_ into H_2_O and O_2_ is spectrophotometrically (Cary 100 UV-VIS, Agilent Technologies, Santa Clara, CA, USA) followed as the decrease at 240 nm and quantified by its molar extinction coefficient (MEC = 36 M^−1^ cm^−1^). A unit of CAT activity is defined as the amount of enzyme that decomposes 1 μmol H_2_O_2_ for 1 min at pH 7.0 and 25 °C. CAT activity was expressed as μmol H_2_O_2_/min/mL.

### 2.7. Data Processing and Statistical Treatment

Data import, manipulation, and analyses were performed within the R environment [31]. The data collected by the various experiments were organized in two master tables: one for the dose–response measures (where the independent variable was dose [Gy]) and another for the recovery measures (where the independent variable was days after irradiation, dai). The datasets were separately analyzed for hypothesis testing and curve fitting. Data importing and manipulation were performed using the tidyverse family of packages [32]. All procedures not detailed in this section were carried out de novo in the code.

#### 2.7.1. Hypothesis Testing

For the former, Kruskal–Wallis tests were conducted separately for each marker to evaluate the significance of overall group differences (based on median and ranges), and Dunn’s tests with Holm adjustment were used for pairwise comparisons of the median. A p-value adjustment was made considering each marker separately. Annotated boxplots were produced using the ggstatsplot::ggbetweenstats() function [33] with type=“Nonparametric”, activating pairwise.comparison and setting p.adjust.method = “holm”. To generate letter-base notation, we applied the same test, posthoc, and p-value adjustment, using a combination of functions from the rstatix package [34] and multcompView::multcompLetters() function [35]. The final plot was produced combining geom_text(), stat_summary() and geom_point() from the ggplot2 package [36]. For recovery measures, we used marker-wise wilcox.test() and p.adjust with method = “holm”, limiting comparisons to (control, day_x) pairs.

To evaluate the correlation between (variable, variable) and (variable, dose or days), we used psych::corr.test() [37] with method = “spearman”, leaving the default (Holm) p-value adjustment. The two master tables were separately evaluated, and the result was displayed using ggplot2 and combining geom_pointrange() and geom_label() layers.

#### 2.7.2. Dose–Response and Recovery Trend Modeling

To model trends in the two datasets, we wrote a custom R script that fits seven equations associated with three functional forms (Table S1) using both standard and nonlinear fitting. Standard fitting was conducted with the minpack.lm::nlsLM() function [38] and 100 iterations (maxit = 100), while robust regression was implemented using the robustbase::nlrob() function [39] with method = “M” and maxit = 400. Robust regression was applied to account for outliers or heteroskedasticity. Initial parameter values for model fitting were dynamically calculated based on the dataset characteristics. Model performance was assessed using adjusted R2, while model selection was carried out based on the Akaike Information Criterion differences (ΔAIC) using a ΔAIC < 2 threshold to select the best model. In case of ties, we selected the simplest function from the group. Diagnostic plots (res vs. fit, normal qqplot, time/order, and scale/location) were produced for the two groups of best fits.

Fits were overimposed on data ranges using stat_summary(), geom_point(), and geom_line() from the ggplot2 package.

## 3. Results

### 3.1. Oxidative Stress and Photosynthetic Markers Under Increasing Radiation Doses

**Figure 1:**
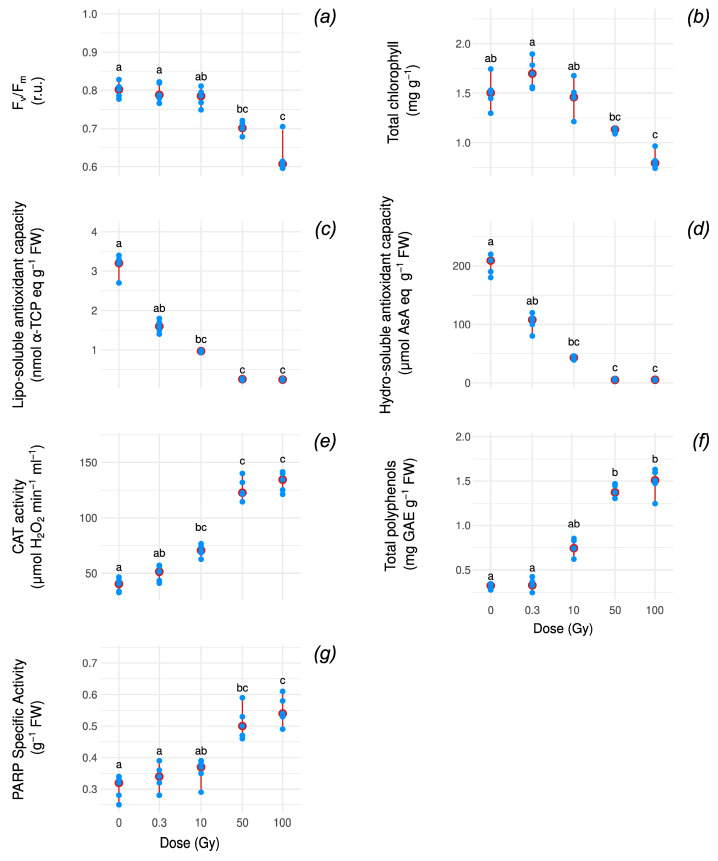
Point measurements (blue), median values (red point), and range (2.5 and 97.2 quartile values, red segment) across doses (x-axis) for each variable: F_v_/F_m_ (**a**), total chlorophyll (**b**), lipo-soluble antioxidant capacity (**c**), hydro-soluble antioxidant capacity (**d**), CAT activity (**e**), total polyphenols (**f**), and PARP specific activity (**g**) in response to increasing doses of X-rays. Letters indicate statistical difference among doses; variables sharing the same letter are not significantly different (Dunn’s test with Holm *p*-value adjustment; see Figure S1 for details). F_v_/F_m_, maximum PSII photochemical efficiency; α-TPC, α-tocopherol; AsA, ascorbic acid; CAT, catalase; GAE, gallic acid equivalent; PARP, poly(ADP-ribose) polymerase activity.

Here, we quantified multiple antioxidant response markers (ARMs), including lipo/hydro-soluble antioxidant activity (lipo/hydro-AOX), catalase activity (CAT), total polyphenol content (POLY), and PARP activity, as well as photosynthetic performance indicators, chlorophyll content (CHL), and maximum PSII quantum yield (F_v_/F_m_), in five *P. vulgaris* plants across a radiation dose range of 0–100 Gy (Figure 1).

Below doses of 10 Gy, photosynthetic performance (CHL and F_v_/F_m_) did not change significantly (Figure 1a,b).

Among the ARMs, lipo- and hydro-AOX levels declined with increasing doses (Figure 1c,d), while CAT, POLY, and PARP exhibited an upward trend (Figure 1e–g).

None of these differences are significant under Kruskal–Wallis (KW) parametric tests followed by Dunn’s test with a Holm-adjusted *p*-value (Figure S1).

In contrast, for doses above 10 Gy, both CHL and F_v_/F_m_ decreased significantly (KW, Holm-adj. *p* ≤ 0.05), with decreases calculated over median values of the control of 50% and 25%, respectively (KW, Holm-adj. *p* ≤ 3 × 10^−2^ and 6.88 × 10^−3^, respectively) (Figure 1a,b and Figure S1).

Concerning ARMs, the changes at doses above 10 Gy were more pronounced; lipo-AOX and hydro-AOX were rapidly and almost completely extinguished (KW, Holm-adj. *p* ≤ 1.43 × 10^−3^, *p* ≤ 1.09 × 10^−3^) (Figure 1c,d and Figure S1), while CAT, POLY, and PARP continued to increase and appeared to plateau at the highest dose tested (KW, Holm-adj. *p* < 0.03) (Figure 1e–g and Figure S1). At the highest dose (100 Gy), the median CAT activity and total polyphenolic content significantly increased three- and fivefold (KW, Holm-adj. *p* ≤ 1.31 × 10^−3^, and 2.01 × 10^−3^, respectively) compared to the control, respectively (Figure 1e,f and Figure S1), while PARP increased by ~69% (KW, Holm-adj. *p* ≤ 3.03 × 10^−3^ (Figure 1g and Figure S1).

For some markers, the final treatments share the same letter, indicating a plateau in the response.

**Figure 2:**
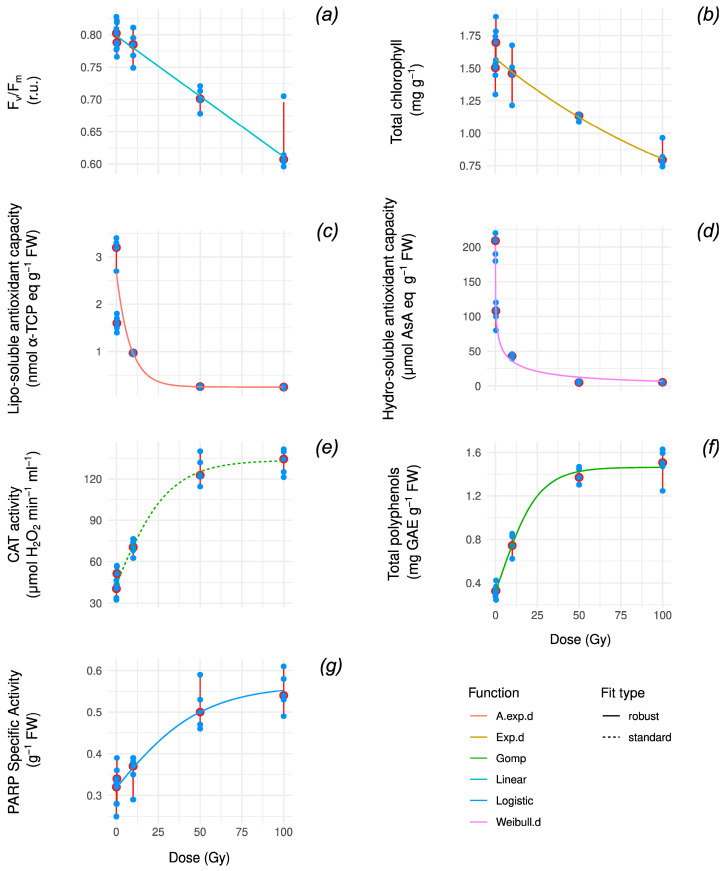
Curve fitting of incremental X-ray dose response for F_v_/F_m_ (**a**), total chlorophyll (**b**), lipo-soluble antioxidant capacity (**c**), hydro-soluble antioxidant capacity (**d**), CAT activity (**e**), total polyphenols (**f**), and PARP specific activity (**g**) overlaid on the same data from Figure 1 (point measurements in blue, median and range in red). Each curve is color coded based on its functional form. F_v_/F_m_, maximum PSII photochemical efficiency; α-TPC, α-tocopherol; AsA, ascorbic acid; CAT, catalase; GAE, gallic acid equivalent; PARP, poly(ADP-ribose) polymerase activity. For each fit, diagnostic plots are reported in Figure S2, while fitted parameter values for best models are reported in Table S3.

To further elucidate these trends, we examined the functional forms that best fit each marke*r’*s response by considering linear, asymptotic, and logistic models and employing robust regression (Figure 2, Appendix A Table A1).

Overall, these data suggest that CHL and F_v_/F_m_ follow approximately linear patterns (Figure 2a,b), whereas the ARMs exhibit approximate asymptotic behavior (Figure 2c,d).

More specifically, CHL is best described by an exponential function (adj. R^2^ = 0.827) whose low decay rate (b = 6.752 × 10^−3^ ) makes it approximately linear (Figure 2a), whereas F_v_/F_m_ exhibits a linear pattern (adj. R^2^ = 0.861) (Figure 2b). By contrast, lipo-AOX and hydro-AOX fit asymptotic curves (adj. R^2^ = 0.978 and 0.752, respectively) (Figure 2c,d) and CAT, PARP, and POLY fit logistic curves (adj. R^2^ = 0.957, 0.829, 0.967) (Figure 2e–g).

Spearman correlation tests confirm the existence of significant and strong correlations between variable pairs (median |ρ| > 0.69, Holm-adj. *p* ≤ 1.33 × 10^−4^) and variable/dose pairs (median |ρ| > 0.8, Holm-adj. *p* ≤ 6.06 × 10^−5^). As expected, across variables, the highest correlations occur between hydro-AOX, lipo-AOX; ρ = 0.935, CI = [0.777, 0.982], Holm-adj. *p* ≤ 4.39 × 10^−8^, PARP, CAT; ρ = 0.877, CI = [0.619, 0.946], Holm-adj = 2.31 × 10^−8^, PARP, POLY; ρ = 0.831, CI = [0.512, 0.949], Holm-adj. *p* ≤ 4.06 × 10^−6^, and CAT, POLY; ρ = 0.866, CI = [0.593,0.960], Holm-adj. *p* ≤ 3.83 × 10^−7^, while the highest anticorrelations occur between F_v_/F_m_, CAT; ρ = 0.821, CI = [−0.945, −0.491], Holm-adj *p* ≤ 1.33 × 10^−4^, lipo/hydro-AOX, POLY, and lipo/hydro-AOX, CAT (Figure S3).

### 3.2. Recovery Dynamic After Exposure to High Doses of X-Ray Radiation

To assess the recovery dynamics after exposure to high doses of radiation, we measured recovery ARMSs and photosynthetic performance indicators (F_v_/F_m_, CHL) on the five plants exposed to 50 Gy.

Pairwise Wilcoxon tests (W) between control conditions and time points are significant (W, Holm-adj. *p* < 0.04, Table S2) for measures taken immediately after irradiation. Photosynthetic performance indicators then recover, while differences are still significant after three days for PARP (W, Holm-adj. *p* < 0.04, Table S2) and in the whole time range for other ARMs (W, Holm-adj. *p* < 0.04, Table S2).

**Figure 3:**
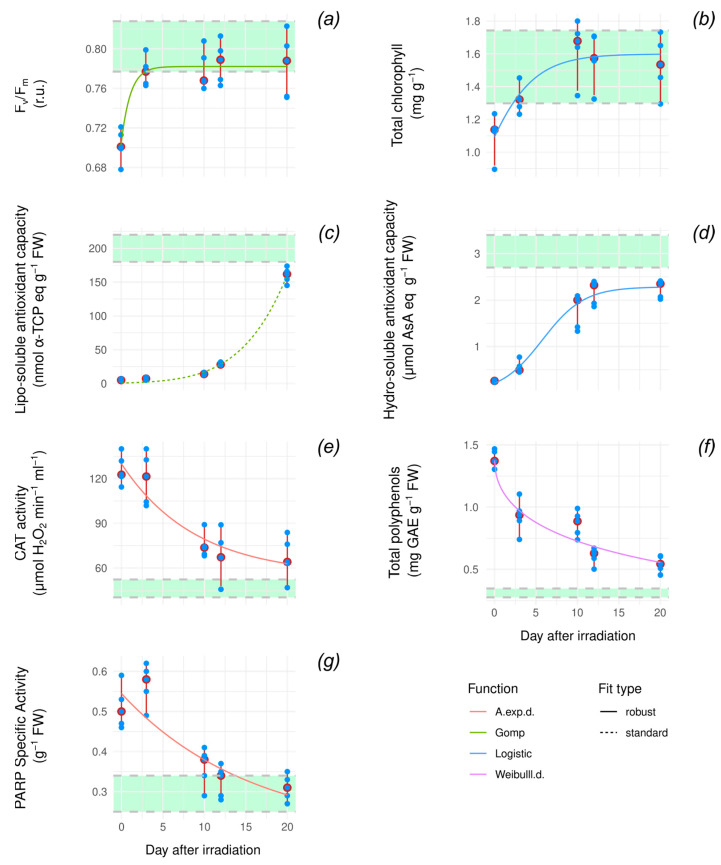
Curve fitting of recovery dynamics for F_v_/F_m_ (**a**), total chlorophyll (**b**), lipo-soluble antioxidant capacity (**c**), hydro-soluble antioxidant capacity (**d**), CAT activity (**e**), total polyphenols (**f**), and PARP specific activity (**g**) soon after a 50 Gy X-ray irradiation event (0), and at 3, 10, 12, and 20 days from irradiation. Curves are color coded based on their functional form. Green area marks control value ranges, with extremes representing the minimum and maximum value measured in control plants for that variable. Days after irradiation are on the x-axis, with 0 marking the moment immediately after irradiation. F_v_/F_m_, maximum PSII photochemical efficiency; α-TPC, α-tocopherol; AsA, ascorbic acid; CAT, catalase; GAE, gallic acid equivalent; PARP, poly(ADP-ribose) polymerase activity. For each fit, diagnostic plots are reported in Figure S5, while fitted parameter values for best models are reported in Table S4. best recovery dynamics curve fittings in Table S4.

As we did for the dose–response analysis, we examined the functional forms that best fit each marker’s response by considering linear, asymptotic, and logistic models and employing robust regression (Figure 3; Table A2). All markers converge toward the control value, albeit at different speeds, meaning that plants recover in 20 days.

Both photosynthetic performance indicators are fit by logistic behavior (logistic function, adj. R^2^ = 0.603 (CHL) and Gompertz function, adj. R^2^ = 0.689 (F_v_/F_m_)). Both display a very early flexus, marking an asymptotic-like recovery (Figure 3a,b). The wide range of values for these indicators considerably affects the R^2^, but it is sufficient to have a first-order approximation of the dynamic. The F_v_/F_m_ fit suggests that efficiency recovers faster than pigment content but fails to return at pre-stress levels. Across ARMs, it is worth noting the different behaviors of lipo/hydro-AOX, with the former fitting an almost exponential Gompertz dynamic (adj. R^2^ = 0.990) and the latter fitting the only clearly logistic curve of the variable set (adj. R^2^ = 0.927) (Figure 3c,d). Both display incomplete recovery after 20 days, and lipo-AOX recovery happens later than every other AOX marker and is abrupt. CAT and POLY are both described by an asymptotic behavior toward recovery (y = 1), but neither seems to recover completely (Figure 3e,f). Both fits display a moderate adj. R^2^ (0.741 and 0.851). For CAT, the data range at three days negatively impacts the fit, decreasing the adj R^2^. Tot poly at ten days is a curve outlier as well.

Lastly, PARP activity displays the simplest behavior, with a linear fit characterized by a low adj. R^2^ (0.681) (Figure 3g).

Spearman correlation tests between variable pairs measured during recovery and variable/days after irradiation pairs often display a wide confidence interval (median value 0.69). The two strongest significant relations are found between POLY and days after irradiation (ρ = −0.914, *p* <= 6.98 × 10^−13^, CI = [−0.976; −0.710]) and lipo-AOX/days after irradiation (ρ = 0.961, *p* <= 7.45 × 10^−7^, CI = [0.860; 0.990]). These two variables also show a strong anticorrelation (ρ = −0.851, *p* <= 7.75 × 10^−4^, CI = [−0.958; −0.537]), as shown in Figure S4.

## 4. Discussion

Our paper identifies a threshold value for the onset of cell damage induced by ionizing radiation (IR), namely, X-rays, delivered on dwarf bean plants at increasing doses. Furthermore, our experiments also analyze the intrinsic capacity of plants to restore from stress imposed by IR monitoring some key antioxidant compounds and enzymes involved in ROS detoxification and DNA repair mechanisms in plant tissue. Oxidative stress in plants is a multifaceted phenomenon that arises when there is an imbalance between the production of reactive oxygen species (ROS) and the plant’s ability to detoxify these reactive intermediates or repair the resulting damage. This condition is commonly triggered by various biotic and abiotic stresses, including pathogen attacks, drought, high salinity, and extreme temperatures [40].

The presence of ROS, mainly hydrogen peroxide (H_2_O_2_), singlet oxygen (^1^O_2_), the superoxide anion radical (O_2_•−), and the hydroxyl radical (OH•), can lead to oxidative damage to essential biomolecules, like lipids, proteins, and nucleic acids, potentially resulting in cellular dysfunction and programmed cell death [41]. Furthermore, the action of radiation-induced ROS is higher in tissues rich in water because IR determines the radiolysis of water molecules, increasing the production of free radicals. It has been demonstrated that elevated doses of IR, even if plants are more radioresistant than animals, may compromise both plant function and structure [7,20]. Scientific evidence also indicates an innate capability by plants to counteract injuries imposed by both high- and low-LET ionizing radiation by means of a plethora of functional, structural, and biochemical defenses [23]. An important parameter in assessing plant response to IR is the dose rate. In our experimental design, despite increasing doses of X-rays, the dose rate is constant and approximately in the range of exposure in high radiation areas after a nuclear disaster. Thus, we can assimilate our setup to acute exposures.

The species *P. vulgaris* used in our study has been chosen as a model in previous research on the impact of ionizing radiation on plants, elucidating the consequences of increasing X-ray dose on aspects related to plant development, photosynthesis, and leaf anatomy and the variability in response due to the aging of irradiated leaves [24,42].

These experiments emphasized the high capability of bean plants to counteract the oxidative stress following plant exposure to high radiation levels and surviving injuries. Here, we aimed to assess the behavior of oxidative defenses and DNA repair systems as dose increases and the kinetics of plant recovery in parallel with the changes in antioxidant and PARP patterns to elucidate their role in plant “radioresilience”. In our experiment, below doses of 10 Gy, photosynthetic performance (CHL and F_v_/F_m_ ratio) did not change significantly compared to irradiated control, suggesting that the functionality of the *P. vulgaris* photosynthetic apparatus remains almost stable. This is unexpected considering that the IR target was the whole plant and not seeds, which is recognized as the most radioresistant plant growth stage [24]. Studies conducted on rats using hematological parameters showed that the dose of 10 Gy of X-rays is much higher than the lethality threshold for animals [43]. The LD50/30 for rats exposed to whole-body irradiation of X-rays through the linear accelerator was found to be 6.6 Gy, while for selected domestic mammals, it was less than 3.5 Gy, with differences among animal species [44].

It is ascertained that the degree of plant damage after exposure to IR also depends on the dose, type of radiation, linear energy transfer (LET), and exposure time (acute or chronic). Plant phenological stage and species’ intrinsic properties are important variables in determining responses [45].

Therefore, the dwarf bean photosynthetic apparatus’s resistance up to a dose of 10 Gy may reasonably be due to a multiple plant cell defense system. Interestingly, differently from photosynthetic endpoints, the ARMs and lipo- and hydro-AOX levels progressively declined up to 10 Gy, while CAT and POLY started to increase. The lipo- and hydro-AOX may be hypothesized to continue the first defense line against oxidative stress. The reduction in lipo- and hydro-antioxidant capacity may be explained by the requirement of scavenger molecules acting in both cytoplasm and membranes to counteract the excess of free radicals and maintain the ROS at concentrations useful for signal purposes. It is noteworthy that ROS also play a crucial role as signaling molecules that modulate plant responses to stress by activating various signaling pathways, including those involving calcium ions and mitogen-activated protein kinases [41,46,47].

In contrast, for doses above 10 Gy, both CHL and F_v_/F_m_ decreased significantly, indicating compromised photosynthetic efficiency.

We may hypothesize that the concentration of ROS downregulated by the first defense line may have activated a second protection level represented, in our case, by polyphenols, CAT, and, finally, PARP [48]. Total polyphenols act as scavengers and stabilize lipid membranes while regulating gene expression related to stress response (they regulate catalase). This could be a response to escalated ROS production. They function as a secondary barrier to stabilize membranes, neutralize radicals, and buffer damage during prolonged or high-intensity stress [49]. Insoluble phenolics are found in cell walls, while soluble phenolics are present within the plant cell vacuoles. It has been demonstrated that phenolic compounds increase in bean plants exposed to strong doses of X-rays, exerting a shielding action against ionizing radiation [42].

In adult *Phaseolus vulgaris* plants, no perturbation in leaf structure was observed, even at the highest level of irradiation [24]; however, photosynthetic activity declined significantly [19]. In our experiment, the linear trend for photosynthetic decline at doses above 10 Gy confirms the idea of cumulative radiation damage at a functional level. However, whether secondary, nonlinear effects (e.g., threshold behaviors, delayed damage) might emerge at higher doses cannot be excluded.

The model fit analysis for dose–response may help to explain the plant defense strategy. More specifically, CHL is best described by an exponential function, whereas F_v_/F_m_ exhibits a linear pattern. Conversely, lipo-AOX and hydro-AOX fit asymptotic curves, while PARP and POLY fit logistic curves.

These nonlinear fits suggest that lipo/hydro-AOX activity rapidly stabilizes (i.e., is nearly extinguished as the dose increases), while other ARMs (POLY, CAT, and PARP) gradually saturate at higher doses, with PARP displaying the slowest progression to saturation. A nonlinear logistic fit indicates that lipo/hydro-AOX activity declines to near-zero levels at relatively low doses (<25 Gy), followed by POLY and then CAT/PARP, which plateau before the highest tested dose.

In contrast, the linear trend of photosynthetic performance indicators suggests that increasing the dose would increase the damage, while the nonlinear trend of ARMs suggests that the organisms are unable to increase the magnitude of the response if the dose increases over 100 Gy.

The anticorrelation between F_v_/F_m_ and CAT and between lipo/hydro-AOX and CAT/POLY is biologically coherent, as oxidative stress often triggers a shift from antioxidant scavenging (e.g., lipo-AOX) to enzymatic responses (e.g., CAT). The results align with the transition from non-enzymatic to enzymatic antioxidant mechanisms under increasing stress.

Our data evidence that intense oxidative stress at doses of 50 and 100 Gy knocks off lipo- and hydro-antioxidant capacity, while CAT becomes upregulated to compensate for the failure of the first defense line. It is reasonable that in these circumstances, the DNA repair systems, mediated by PARP, are activated in dwarf beans, marking a multi-layered defense strategy against oxidative stress and damaged DNA.

The increase in PARP activity likely marks a shift from antioxidant de-toxification to DNA-level protection [50].

Furthermore, given PARP’s dependence on NAD+, the increase in PARP activity could mark a two-level metabolic shift from growing to damage control and from antioxidant defense to DNA repair. If too much NAD+ is consumed, metabolic collapse could occur [51].

The kinetics of recovery of photosynthetic performance indicators (CHL and F_v_/F_m_) and antioxidant response markers (ARMs) following the X-ray strong dose of 50 Gy revealed that the multi-layered defense strategy against oxidative stress and damaged DNA was effective, allowing plants to survive. Furthermore, the recovery dynamics associated with different ARMs clarified their contribution to dwarf bean radioprotection over time.

The F_v_/F_m_ ratio at 3 days from irradiation retrieved values near 0.8 considered for the plants in healthy status and remained at a plateau for the following 20 days of observation [52]. Differently, the total chlorophyll pool requires 10 days for a reprise to control values. These temporal differences likely indicate a diverse radiosensitivity of the photosynthetic apparatus components at very high doses of ionizing radiation. It may be hypothesized that light-harvesting complexes are more injured than photosystem II (PSII), requiring further recovery time.

After 3 days from irradiation, CAT and PARP maintain very high activity, indicating their strong involvement in photosynthetic apparatus reprise and pigment pool restoration. We suppose that the oxidative stress and DNA alterations responsible for the sudden photosynthetic decline soon after an acute X-ray dose of 50 Gy persist over time, and a detoxification action is still required to avoid permanent damage to plant cells [14]. Moreover, the increased levels of total polyphenol observed at 10 days post-50 Gy X-ray exposure suggest that their active role in shielding cells from radiation continues to be effective, ensuring radioprotection even after acute exposure [42,53].

The kinetics of CAT, PARP, and polyphenols appear similar and provide evidence of how the role of these antioxidants is important in the first phase of recovery and declines at 20 days after irradiation when they approach respective control values. The return of PARP activity to values close to those measured for not irradiated plants indicates the replacement of intracellular energy levels and DNA damage repair, conditions essential for plant survival [54]. Conversely, the lipo- and hydro-antioxidant capacity increases over time, with different kinetics. Interestingly, the lipo-soluble antioxidant capacity follows an exponential kinetics, while the hydro-soluble antioxidant capacity is a saturation one, even if none come back to control values at pre-stress conditions. It implies that the free radical scavenger system does not eliminate ROS at all, but part of it remains to serve as a messenger in case of stress returning, improving oxidative stress resilience in plants [48].

Our data indicate that in the late phase of recovery from a high dose of acute radiation, the levels of lipo- and hydro-antioxidant pools contribute to maintaining ROS at concentrations for signal purposes that are not dangerous for cells [55].

The strong negative correlation between POLY/days after irradiation and lipo-AOX/days after irradiation indicates that in the late phase of recovery, with the replacement of pre-stress conditions, there is no need for membrane shielding, but there is a requirement for cell detoxification. The anticorrelation also reveals that polyphenols do not contribute to the lipo-antioxidant pool in the late phase.

## 5. Conclusions

Our results demonstrated that the photosynthetic apparatus of dwarf bean plants, irradiated at the vegetative stage with acute X-rays, maintains its functionality up to 10 Gy and is strongly impacted at the high doses of 50 and 100 Gy. In response to increasing doses of X-rays, a plethora of antioxidant defenses are activated to counteract radiation-induced oxidative stress. Our data indicate that intense oxidative stress at doses of 50 and 100 Gy depletes both lipo- and hydro-antioxidant capacities. Concurrently, the activities of catalase and poly(ADP-ribose) polymerase are upregulated, underscoring a multi-layered defense strategy against oxidative stress and damaged DN.

The kinetics of recovery over time, as measured by photosynthetic performance indicators and antioxidant response markers following exposure to an acute dose of 50 Gy, suggest that dwarf bean plants can endure high levels of ionizing radiation, returning to nearly pre-stress conditions in terms of photosynthetic apparatus functionality within just 10 days post-irradiation. Our findings indicate that the multi-layered defense strategy against oxidative stress and DNA damage is effective in enabling plant survival. We observed that PARP, polyphenols, and catalase are particularly active during the initial phase of recovery. In contrast, during the later phase, lipo- and hydro-antioxidant levels help maintain reactive oxygen species (ROS) at concentrations that are safe for the cells. The restoration of pre-stress conditions following severe irradiation highlights the radioresilience of dwarf beans and provides evidence of the crucial role played by antioxidant patterns and PARP in conferring this trait to the plants. Assessing plant resilience capability to ionizing radiation is vital in the context of radiation-contaminated environments, but it is also useful in the view of future space-manned missions where plants will be important components of space platforms.

## Data Availability

Data are available from the corresponding author upon reasonable request.

## Supplementary Materials

The following supporting information can be downloaded at https://www.mdpi.com/article/10.3390/antiox14030261/s1, Figure S1: multi-page detailed hypothesis testing of dose-response effects on the seven markers; Figure S2: multi-page diagnostic plots for non-linear least squares (nls) model fits of seven markers vs radiation dose; Figure S3: correlation analysis for all pairwise comparisons among dose levels and response markers; Figure S4: correlation analysis for all pairwise comparisons among daily recovery points (dai, days after irradiation) and response markers; Figure S5: multi-page diagnostic plots for non-linear least squares (nls) model fits of seven markers vs daily recovery points (dai, days after irradiation). Table S1: master equation table; Table S2: pairwise hypothesis testing for differences between control values and recovery time points across variables; Table S3: fitted parameters value for best dose/response dynamics curve fittings; Table S4: fitted parameters value for best recovery dynamics curve fittings.

## Appendix A

**Table A1:** Evaluation metrics for dose–response dynamics curve fitting. Variable, the name of the variable; Function, function name; Fit type, the type of fit; AIC, Akaike Information Criteria metric; BIC, Bayesian Information Criteria metric; delta_AIC, the difference between AIC minimum for that variable and AIC for that function/row. A.exp.d, asymptotic exponential decay; Exp, exponential; Weibull.d, Weibull decay.

Variable	Function	Fit Type	AIC	BIC	Delta_AIC
F_v_/F_m_	Linear	robust	−117.01	−113.35	0.000
Total chlorophylls	Exp. decay	robust	−51.49	−47.83	0.000
Hydro-AOX	A.exp.d	robust	−27.79	−22.92	0.000
Lipo-AOX	Weibull.d	robust	188.61	193.49	0.000
Catalase	Gompertz	standard	180.10	184.97	0.115
Total polyphenols	Gompertz	robust	−54.64	−49.77	0.000
PARP	Logistic	robust	−85.31	−80.44	0.464

**Table A2:** Evaluation metrics for recovery dynamics curve fitting. Variable, name of the variable; Function, function name; Fit type, type of fit; AIC, Akaike Information Criteria metric; BIC, Bayesian Information Criteria metric; delta_AIC, the difference between AIC minimum for that variable and AIC for that function/row. A.exp.d, asymptotic exponential decay; Exp, exponential; Weibull.d, Weibull decay.

Variable	Function	Fit Type	AIC	BIC	Delta_AIC
F_v_/F_m_	Gompertz	robust	−119.52	−114.65	0
Total chlorophylls	Logistic	robust	−21.99	−17.11	0
Hydro-AOX	Logistic	robust	−6.25	−1.38	0
Lipo-AOX	Gompertz	standard	165.49	170.37	0
Catalase	A.exp.d	robust	205.02	209.89	0
Total polyphenols	Weibull.d	robust	−38.53	−33.66	0
PARP	Gompertz	robust	−66.44	−61.56	0
