# Supplementary material for: Antioxidant Defenses and Poly(ADP-Ribose) Polymerase (PARP) Activity Provide “Radioresilience” Against Ionizing Radiation-Induced Stress in Dwarf Bean Plants
Source: Antioxidants (Basel). 2025 Feb 25;14(3):261. doi: 10.3390/antiox14030261 (PMC11939814; doi:10.3390/antiox14030261)
Supplement: Supplementary file 1 [file antioxidants-14-00261-s001.zip › Figures Caption.pdf]

Figure S1. Multi-page detailed hypothesis testing of dose-response effects on seven markers — F_v/F_m, total chlorophylls, hydro-AOX, lipo-AOX, catalase, total polyphenols, and PARP — across five dose levels (0, 0.3, 10, 50, and 100) with n=5 per group. Data are presented as boxplots, and statistical analyses were performed using the Kruskal–Wallis test with Dunn's post hoc comparisons (Holm-adjusted p-values) and ordinal effect sizes (ε ordinal) with 95% confidence intervals.

Figure S2. Multi-page diagnostic plots for non-linear least squares (nls) model fits of seven markers vs radiation dose — F_v/F_m (nls lin), total chlorophylls (nls exp), hydro-AOX (nls asym), lipo-AOX (nls wdec), catalase (nls gomp), total polyphenols (nls gomp), and PARP (nls logi). For each marker, diagnostic plots (Residuals vs Fitted, Normal Q–Q, Residuals vs Index, and Scale–Location) assess model assumptions and fit quality.

Figure S3. Correlation analysis for all pairwise comparisons among dose levels (Dose) and response markers— F_v/F_m, total chlorophylls (Ttlch), hydro-AOX (hyAOX), lipo-AOX (lpAOX), catalase (Catls), total polyphenols (TtlPl), and PARP. Each cell displays the correlation coefficient (ϱ) with its confidence interval and the corresponding color-coded log-transformed, Holm-adjusted p-value

Figure S4. Correlation analysis for all pairwise comparisons among daily recovery points (dai, days after irradiation) and response markers — F_v/F_m, total chlorophylls (Ttlch), hydro-AOX (hyAOX), lipo-AOX (lpAOX), catalase (Catls), total polyphenols (TtlPl), and PARP. Each cell reports the correlation coefficient (ϱ) with its confidence interval and the corresponding color-coded log-transformed.

Figure S5. Multi-page diagnostic plots for non-linear least squares (nls) model fits of seven markers vs daily recovery points (dai, days after irradiation) — F_v/F_m (nls lin), total chlorophylls (nls exp), hydro-AOX (nls asym), lipo-AOX (nls wdec), catalase (nls gomp), total polyphenols (nls gomp), and PARP (nls logi). For each marker, diagnostic plots (Residuals vs Fitted, Normal Q–Q, Residuals vs Index, and Scale–Location) assess model assumptions and fit quality.
