# Supplementary material for: Antioxidant Defenses and Poly(ADP-Ribose) Polymerase (PARP) Activity Provide “Radioresilience” Against Ionizing Radiation-Induced Stress in Dwarf Bean Plants
Source: Antioxidants (Basel). 2025 Feb 25;14(3):261. doi: 10.3390/antiox14030261 (PMC11939814; doi:10.3390/antiox14030261)
Supplement: Supplementary file 1 [file antioxidants-14-00261-s001.zip › Figure_S1.pdf]

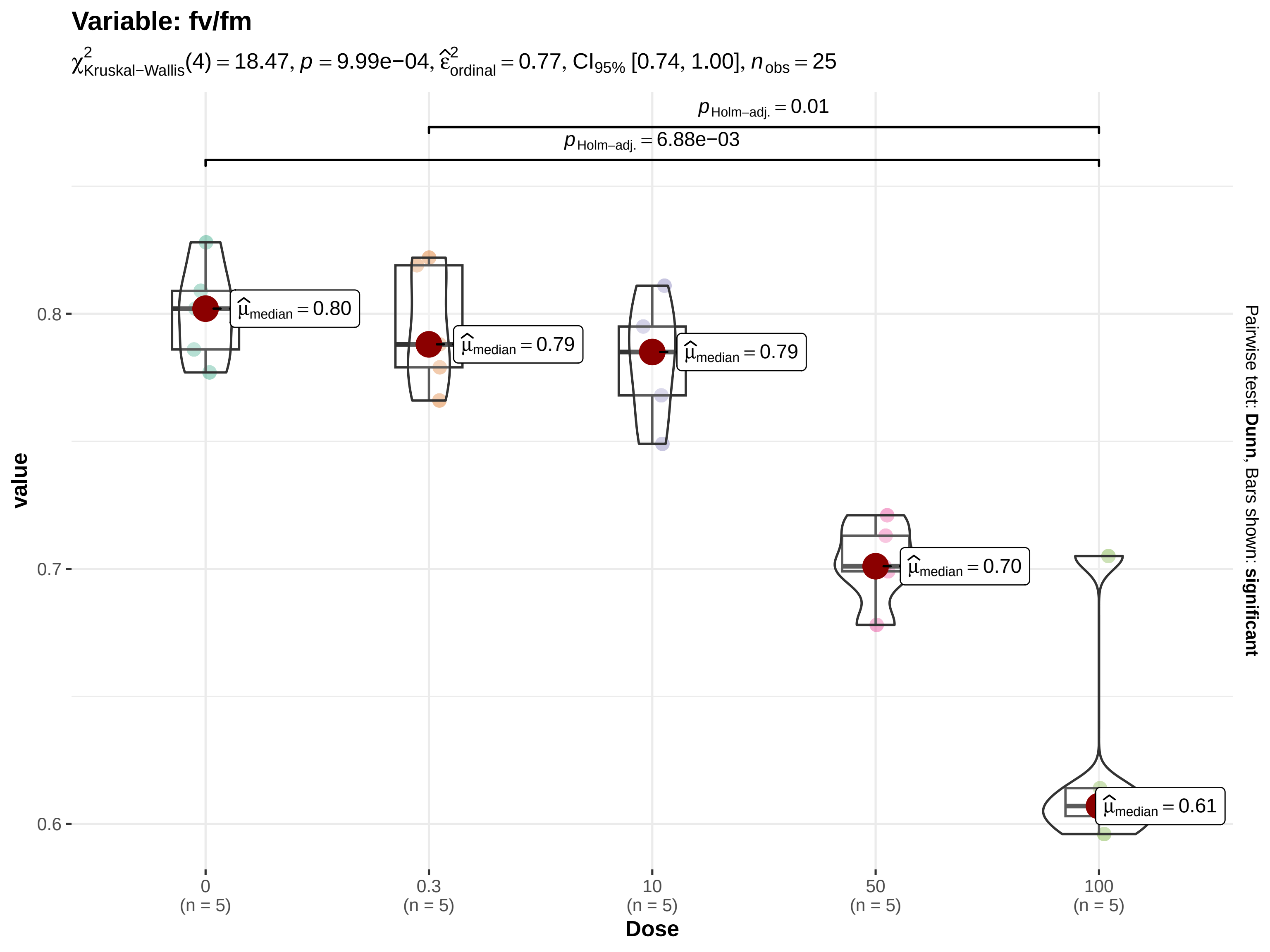

Variable: fv/fm
$\chi^2_{\text{Kruskal-Wallis}}(4) = 18.47$, $p = 9.99\text{e}-04$, $\hat{\varepsilon}^2_{\text{ordinal}} = 0.77$, $\text{CI}_{95\%}$ [0.74, 1.00], $n_{\text{obs}} = 25$
$p_{\text{Holm-adj.}} = 0.01$
$p_{\text{Holm-adj.}} = 6.88\text{e}-03$
$\hat{\mu}_{\text{median}} = 0.80$
$\hat{\mu}_{\text{median}} = 0.79$
$\hat{\mu}_{\text{median}} = 0.79$
$\hat{\mu}_{\text{median}} = 0.70$
$\hat{\mu}_{\text{median}} = 0.61$
value
0.8
0.7
0.6
0
(n = 5)
0.3
(n = 5)
10
(n = 5)
50
(n = 5)
100
(n = 5)
Dose
Pairwise test: **Dunn**, Bars shown: **significant**

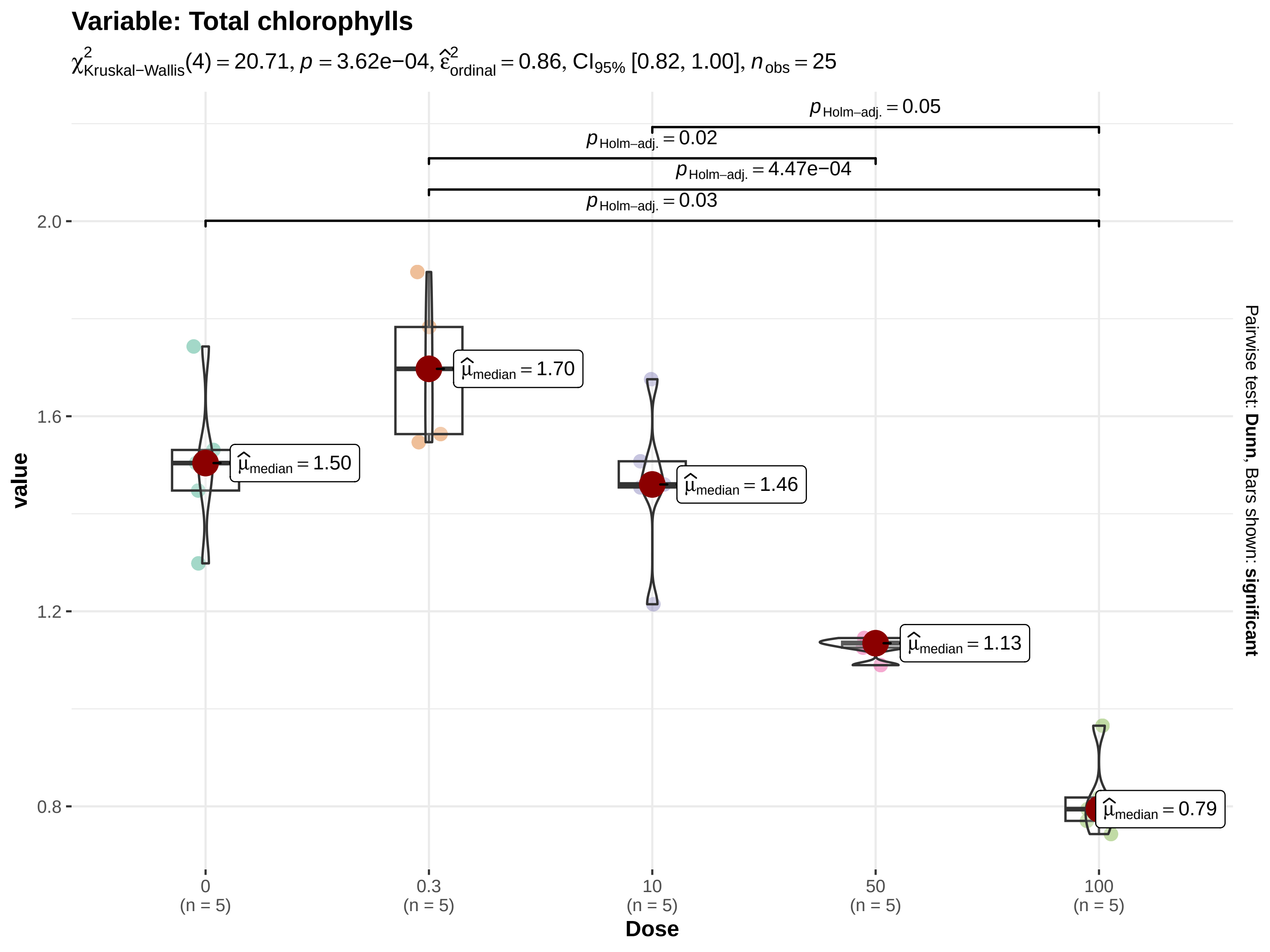
Variable: Total chlorophylls
χ²Kruskal−Wallis(4) = 20.71, p = 3.62e−04, ε̂²ordinal = 0.86, CI95% [0.82, 1.00], nobs = 25
pHolm−adj. = 0.05
pHolm−adj. = 0.02
pHolm−adj. = 4.47e−04
pHolm−adj. = 0.03
μ̂median = 1.50
μ̂median = 1.70
μ̂median = 1.46
μ̂median = 1.13
μ̂median = 0.79
value
2.0
1.6
1.2
0.8
0
(n = 5)
0.3
(n = 5)
10
(n = 5)
50
(n = 5)
100
(n = 5)
Dose
Pairwise test: Dunn, Bars shown: significant

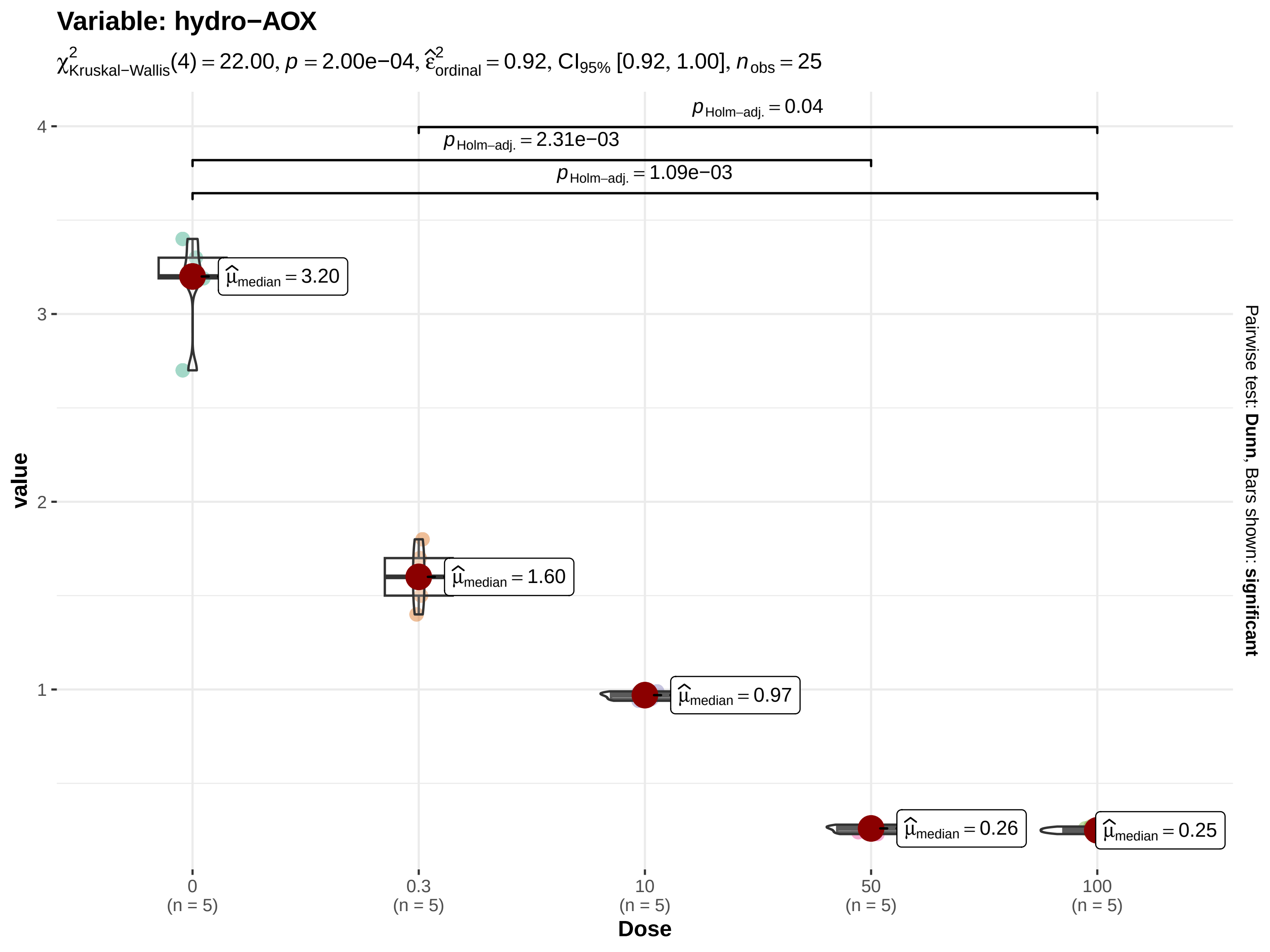

Variable: hydro−AOX
$\chi^2_{\text{Kruskal-Wallis}}(4) = 22.00$, $p = 2.00\text{e}{-04}$, $\hat{\varepsilon}^2_{\text{ordinal}} = 0.92$, $\text{CI}_{95\%}$ [0.92, 1.00], $n_{\text{obs}} = 25$
$p_{\text{Holm-adj.}} = 0.04$
$p_{\text{Holm-adj.}} = 2.31\text{e}{-03}$
$p_{\text{Holm-adj.}} = 1.09\text{e}{-03}$
$\hat{\mu}_{\text{median}} = 3.20$
$\hat{\mu}_{\text{median}} = 1.60$
$\hat{\mu}_{\text{median}} = 0.97$
$\hat{\mu}_{\text{median}} = 0.26$
$\hat{\mu}_{\text{median}} = 0.25$
value
4
3
2
1
0
(n = 5)
0.3
(n = 5)
10
(n = 5)
50
(n = 5)
100
(n = 5)
Dose
Pairwise test: **Dunn**, Bars shown: **significant**

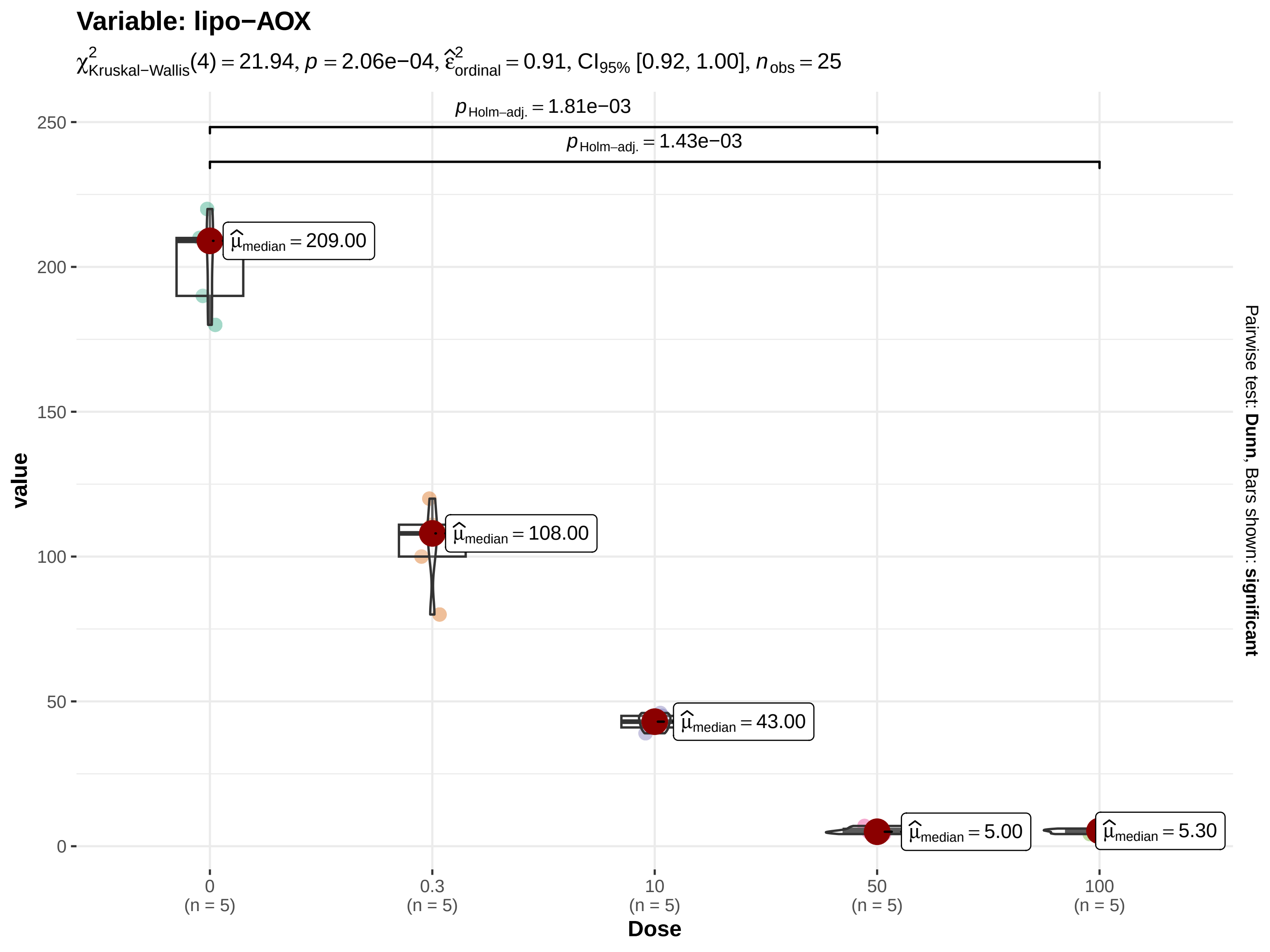

Variable: lipo−AOX
$\chi^2_{\text{Kruskal-Wallis}}(4) = 21.94$, $p = 2.06e-04$, $\hat{\varepsilon}^2_{\text{ordinal}} = 0.91$, $\text{CI}_{95\%}$ [0.92, 1.00], $n_{\text{obs}} = 25$
$p_{\text{Holm-adj.}} = 1.81e-03$
$p_{\text{Holm-adj.}} = 1.43e-03$
$\hat{\mu}_{\text{median}} = 209.00$
$\hat{\mu}_{\text{median}} = 108.00$
$\hat{\mu}_{\text{median}} = 43.00$
$\hat{\mu}_{\text{median}} = 5.00$
$\hat{\mu}_{\text{median}} = 5.30$
value
250
200
150
100
50
0
0 (n = 5)
0.3 (n = 5)
10 (n = 5)
50 (n = 5)
100 (n = 5)
Dose
Pairwise test: **Dunn**, Bars shown: **significant**

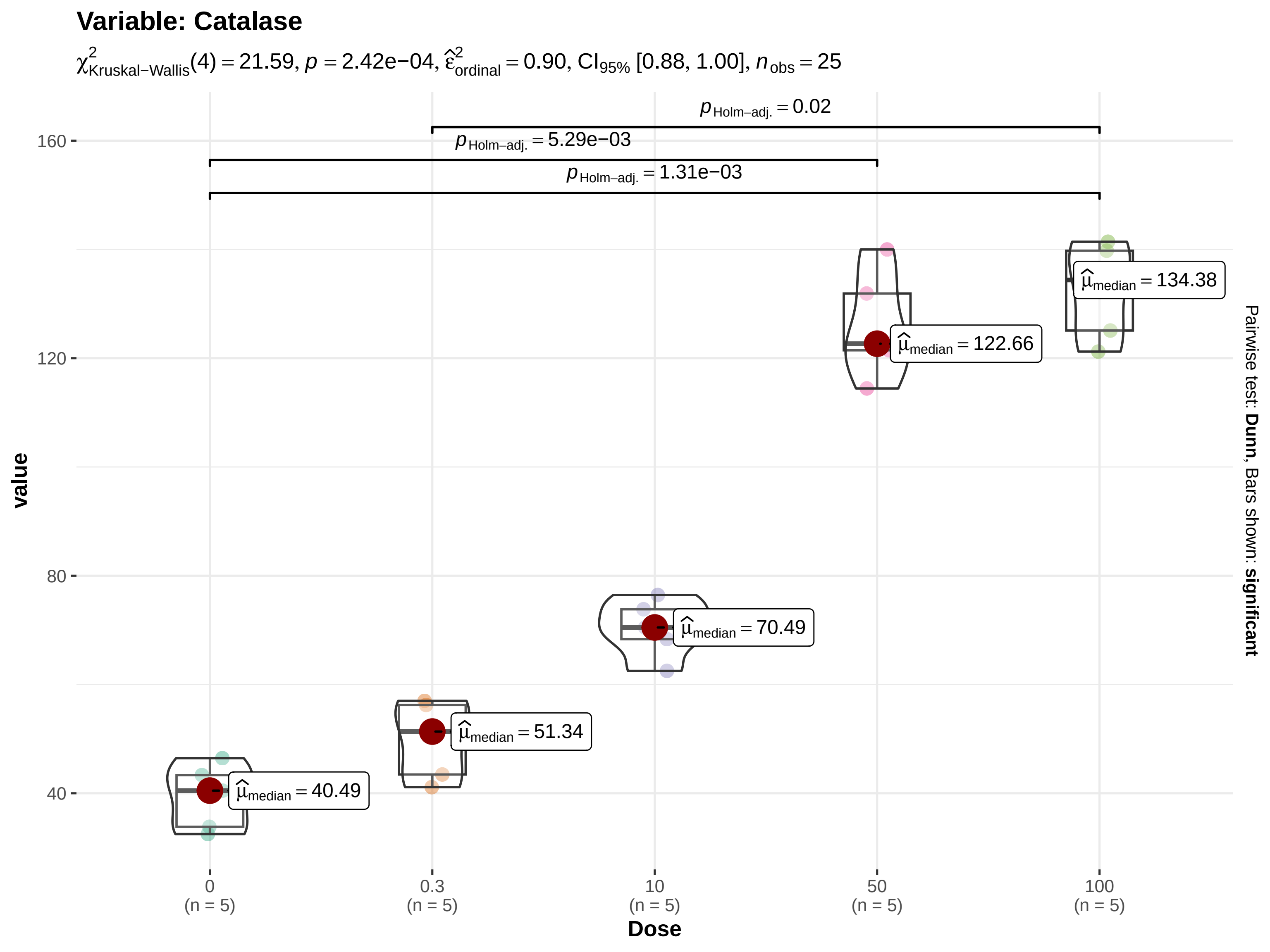
Variable: Catalase
$\chi^2_{Kruskal-Wallis}(4) = 21.59, p = 2.42e-04, \hat{\varepsilon}^2_{ordinal} = 0.90, CI_{95\%}$ [0.88, 1.00], $n_{obs} = 25$
$p_{Holm-adj.} = 0.02$
$p_{Holm-adj.} = 5.29e-03$
$p_{Holm-adj.} = 1.31e-03$
$\hat{\mu}_{median} = 134.38$
$\hat{\mu}_{median} = 122.66$
$\hat{\mu}_{median} = 70.49$
$\hat{\mu}_{median} = 51.34$
$\hat{\mu}_{median} = 40.49$
160
120
80
40
value
0 (n = 5)
0.3 (n = 5)
10 (n = 5)
50 (n = 5)
100 (n = 5)
Dose
Pairwise test: Dunn, Bars shown: significant

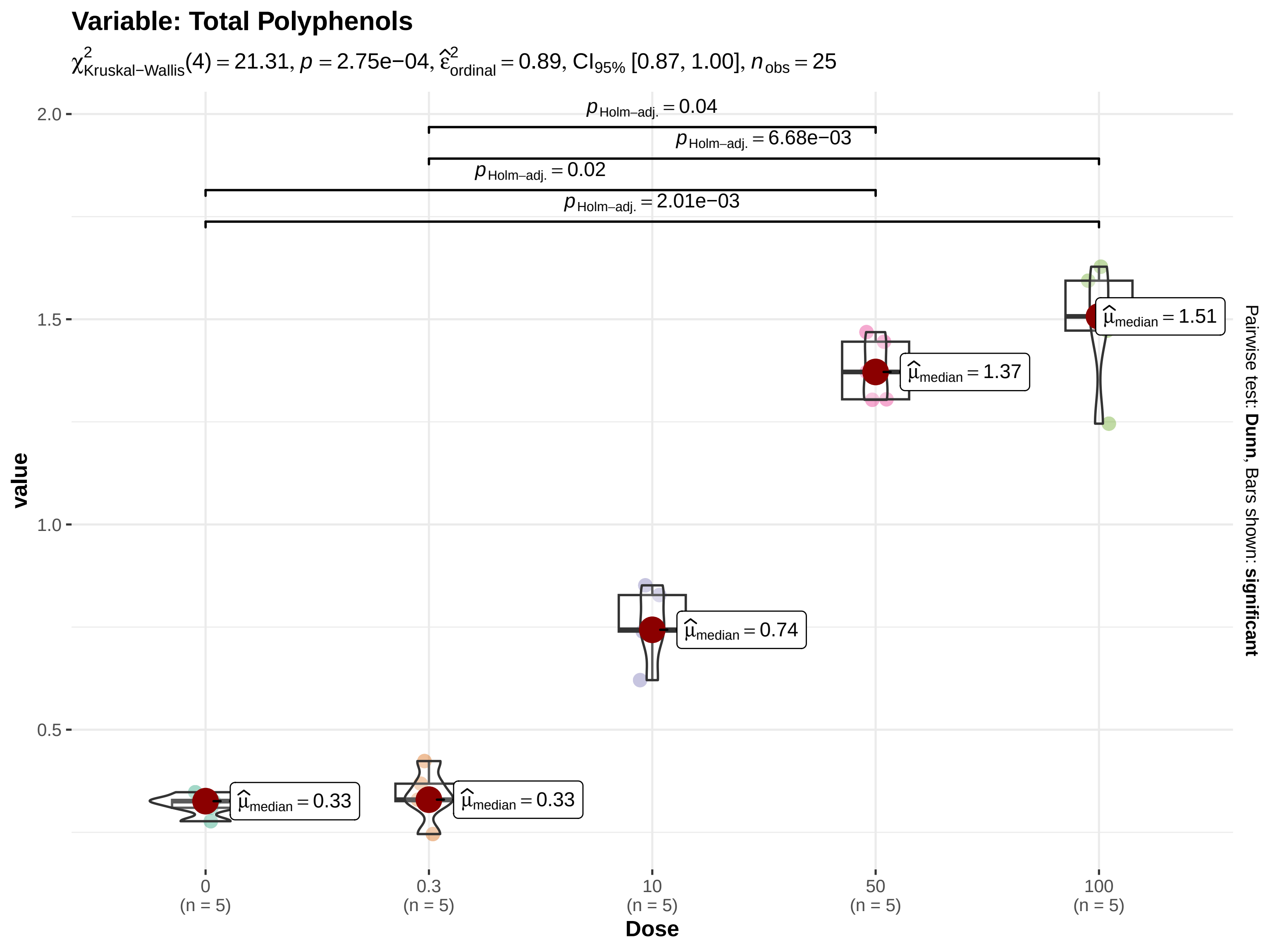

Variable: Total Polyphenols
$\chi^2_{\text{Kruskal-Wallis}}(4) = 21.31$, $p = 2.75\text{e}{-04}$, $\hat{\varepsilon}^2_{\text{ordinal}} = 0.89$, $\text{CI}_{95\%}$ [0.87, 1.00], $n_{\text{obs}} = 25$
$p_{\text{Holm-adj.}} = 0.04$
$p_{\text{Holm-adj.}} = 6.68\text{e}{-03}$
$p_{\text{Holm-adj.}} = 0.02$
$p_{\text{Holm-adj.}} = 2.01\text{e}{-03}$
$\hat{\mu}_{\text{median}} = 0.33$
$\hat{\mu}_{\text{median}} = 0.33$
$\hat{\mu}_{\text{median}} = 0.74$
$\hat{\mu}_{\text{median}} = 1.37$
$\hat{\mu}_{\text{median}} = 1.51$
value
2.0
1.5
1.0
0.5
0
(n = 5)
0.3
(n = 5)
10
(n = 5)
50
(n = 5)
100
(n = 5)
Dose
Pairwise test: **Dunn**, Bars shown: **significant**

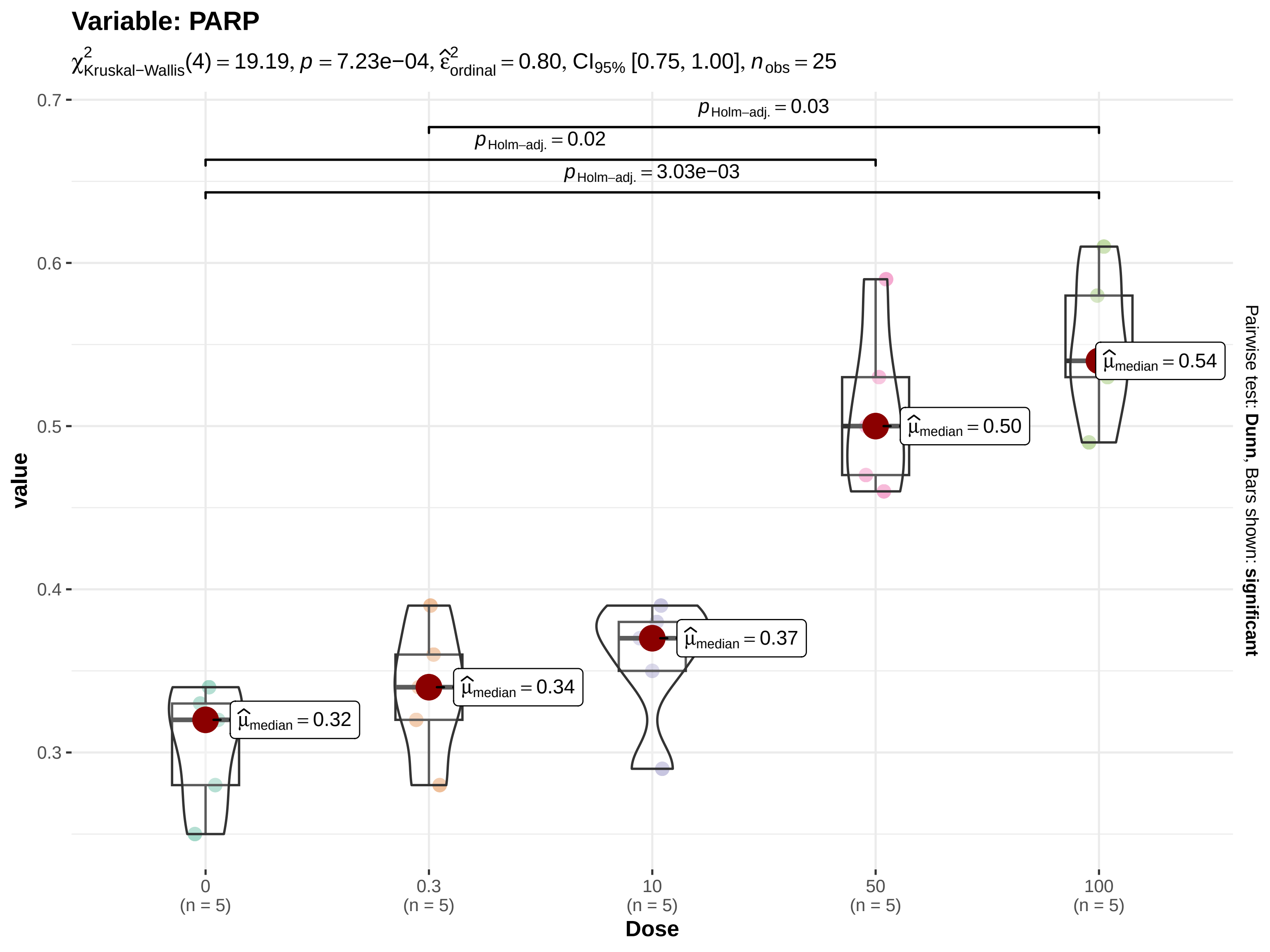
Variable: PARP
$\chi^2_{Kruskal-Wallis}(4) = 19.19$, $p = 7.23e-04$, $\hat{\varepsilon}^2_{ordinal} = 0.80$, $CI_{95\%}$ [0.75, 1.00], $n_{obs} = 25$
$p_{Holm-adj.} = 0.03$
$p_{Holm-adj.} = 0.02$
$p_{Holm-adj.} = 3.03e-03$
$\hat{\mu}_{median} = 0.32$
$\hat{\mu}_{median} = 0.34$
$\hat{\mu}_{median} = 0.37$
$\hat{\mu}_{median} = 0.50$
$\hat{\mu}_{median} = 0.54$
value
0.7
0.6
0.5
0.4
0.3
0
(n = 5)
0.3
(n = 5)
10
(n = 5)
50
(n = 5)
100
(n = 5)
Dose
Pairwise test: **Dunn**, Bars shown: **significant**
