# Supplementary material for: Antioxidant Defenses and Poly(ADP-Ribose) Polymerase (PARP) Activity Provide “Radioresilience” Against Ionizing Radiation-Induced Stress in Dwarf Bean Plants
Source: Antioxidants (Basel). 2025 Feb 25;14(3):261. doi: 10.3390/antiox14030261 (PMC11939814; doi:10.3390/antiox14030261)
Supplement: Supplementary file 1 [file antioxidants-14-00261-s001.zip › Figure_S2.pdf]

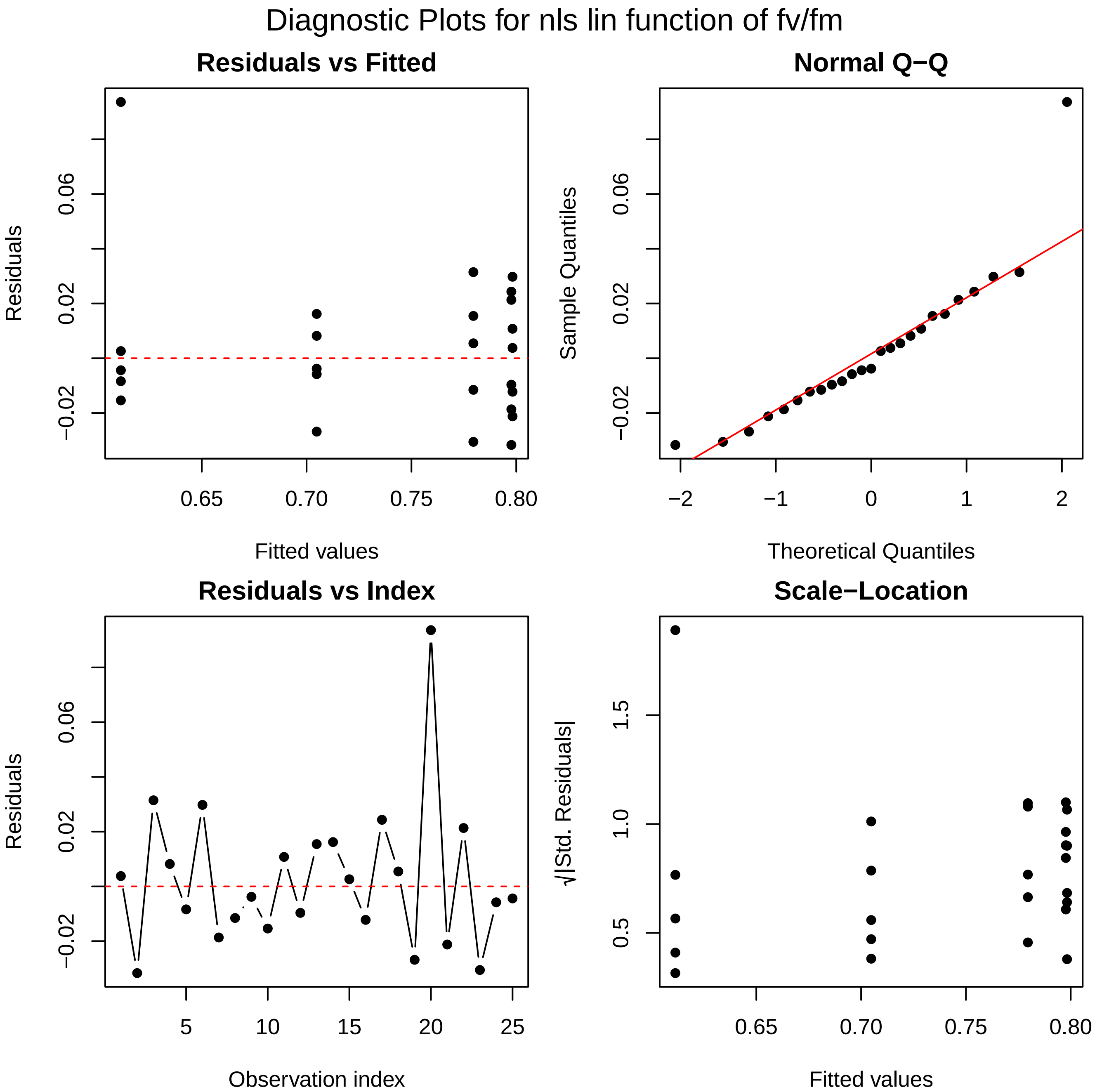
Diagnostic Plots for nls lin function of fv/fm
Residuals vs Fitted
Residuals
0.06
0.02
−0.02
0.65
0.70
0.75
0.80
Fitted values
Normal Q−Q
Sample Quantiles
−2
−1
0
1
2
Theoretical Quantiles
Residuals vs Index
5
10
15
20
25
Observation index
Scale−Location
√|Std. Residuals|
1.5
1.0
0.5
Fitted values

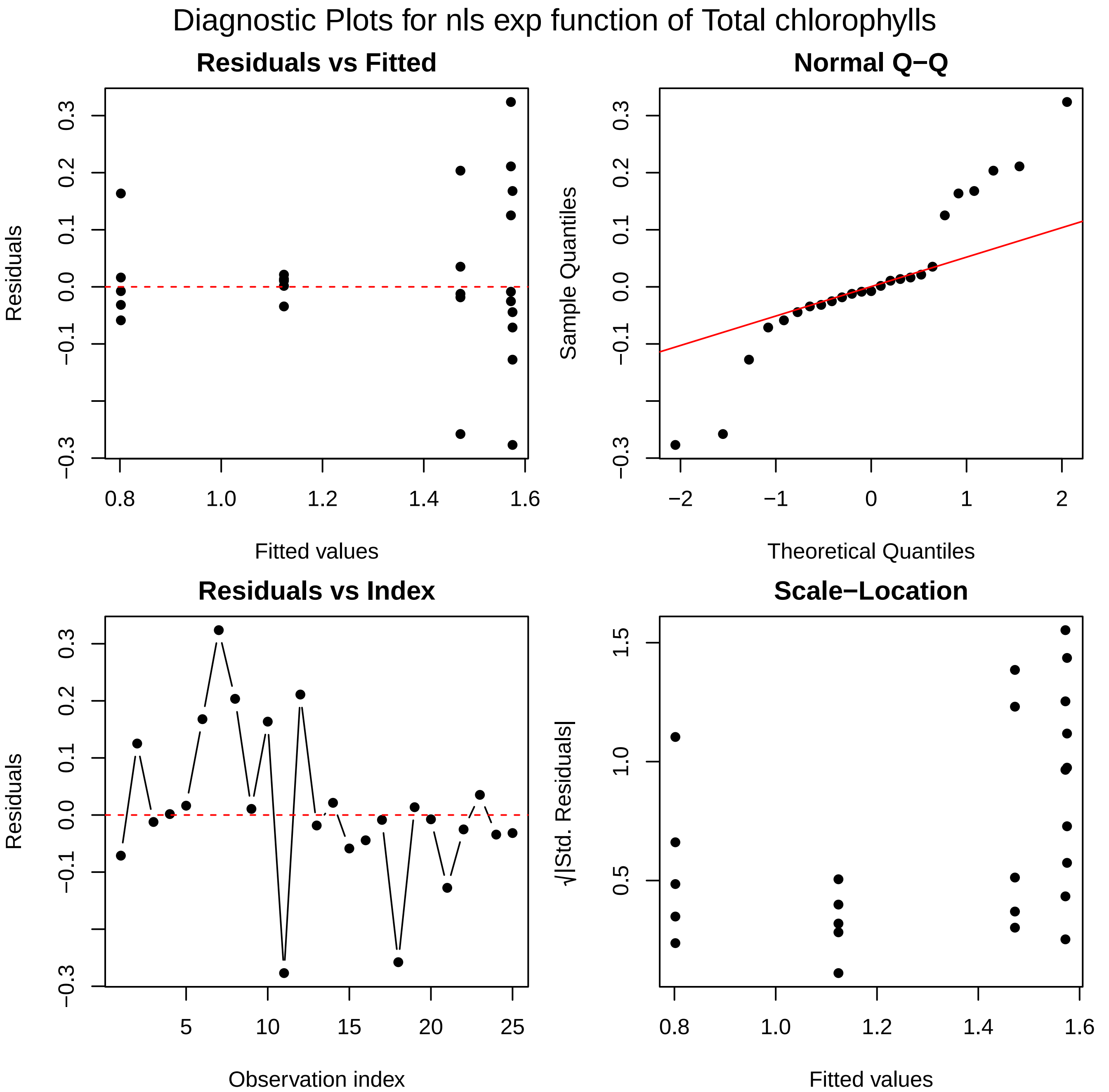
Diagnostic Plots for nls exp function of Total chlorophylls
Residuals vs Fitted
Residuals
Fitted values
0.3
0.2
0.1
0.0
−0.1
−0.3
0.8
1.0
1.2
1.4
1.6
Normal Q−Q
Sample Quantiles
Theoretical Quantiles
−2
−1
0
1
2
Residuals vs Index
Observation index
5
10
15
20
25
Scale−Location
√|Std. Residuals|
1.5
1.0
0.5

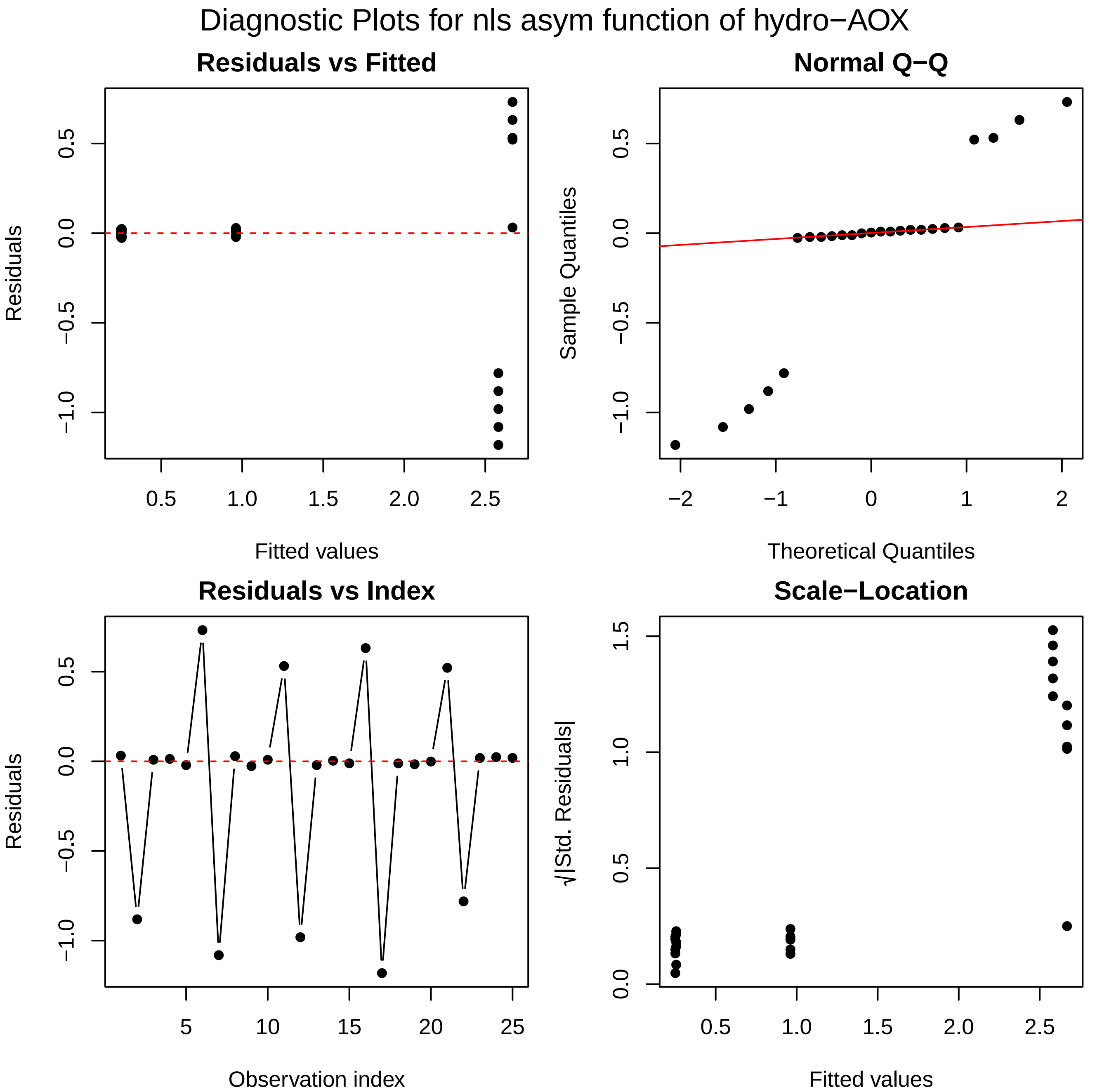
Diagnostic Plots for nls asym function of hydro−AOX
Residuals vs Fitted
Normal Q−Q
Residuals vs Index
Scale−Location
Residuals
Sample Quantiles
√|Std. Residuals|
Fitted values
Theoretical Quantiles
Observation index

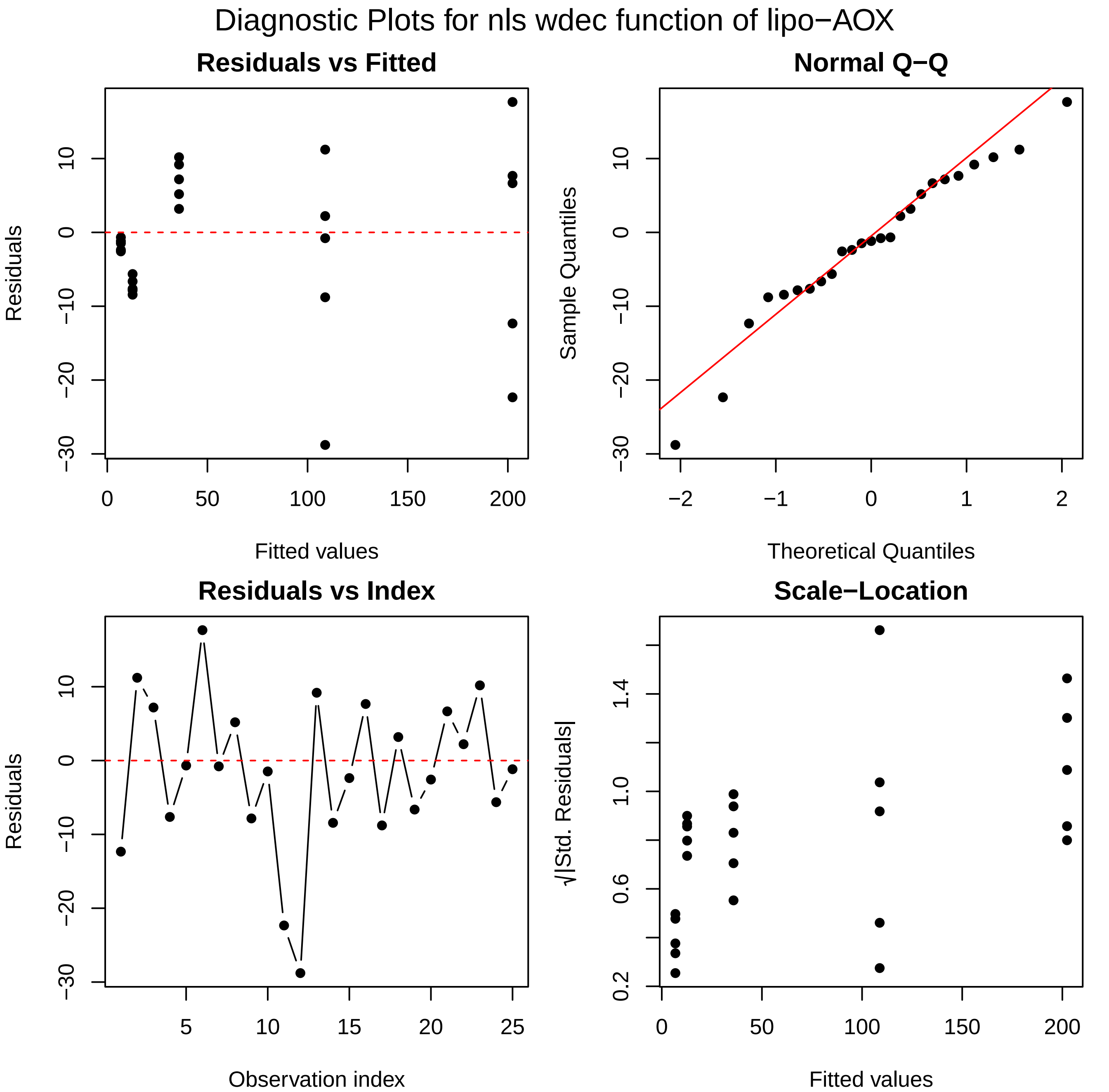
Diagnostic Plots for nls wdec function of lipo−AOX
Residuals vs Fitted
Residuals
Fitted values
Normal Q−Q
Sample Quantiles
Theoretical Quantiles
Residuals vs Index
Residuals
Observation index
Scale−Location
√|Std. Residuals|
Fitted values

Diagnostic Plots for nls gomp function of Catalase

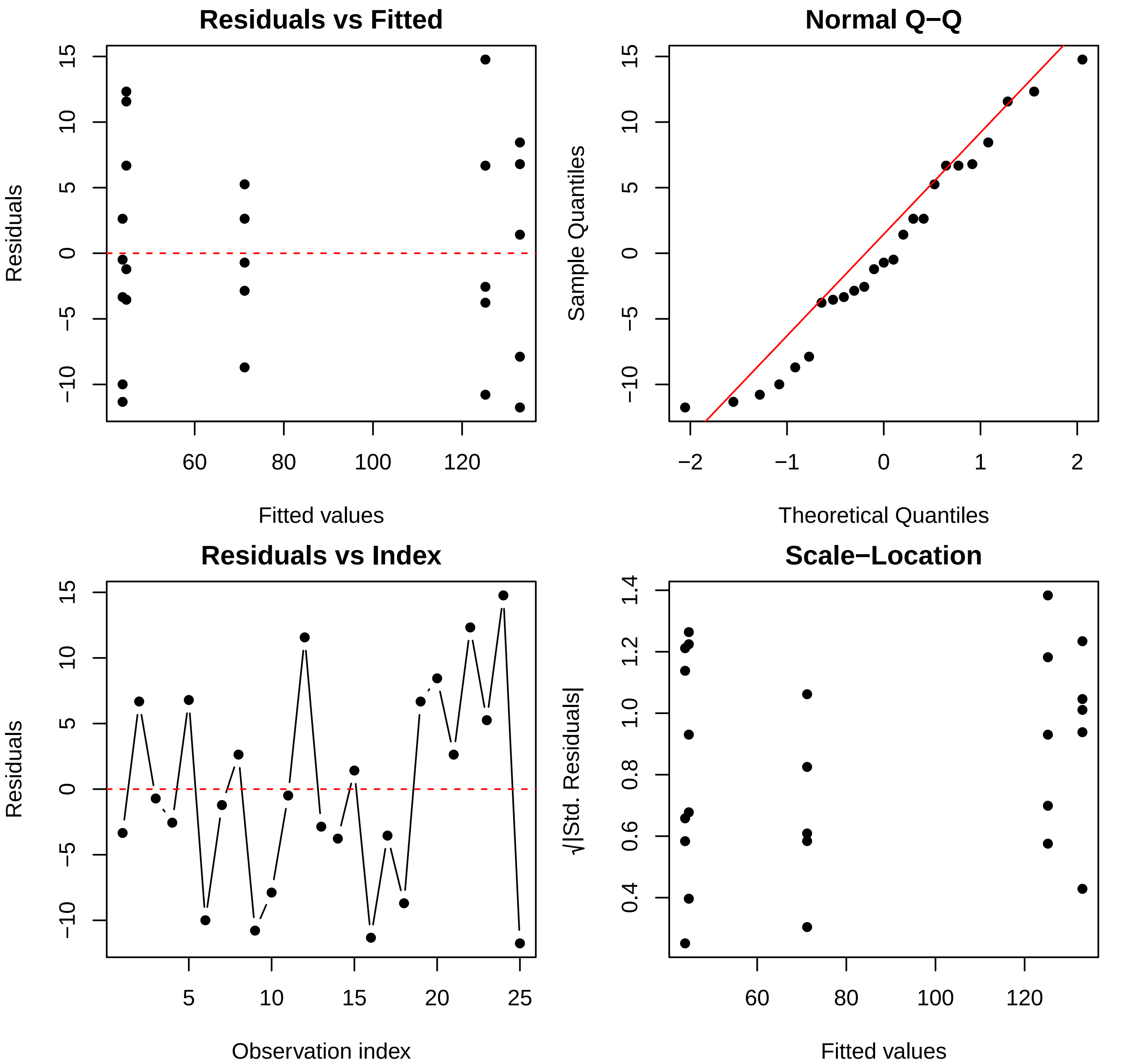

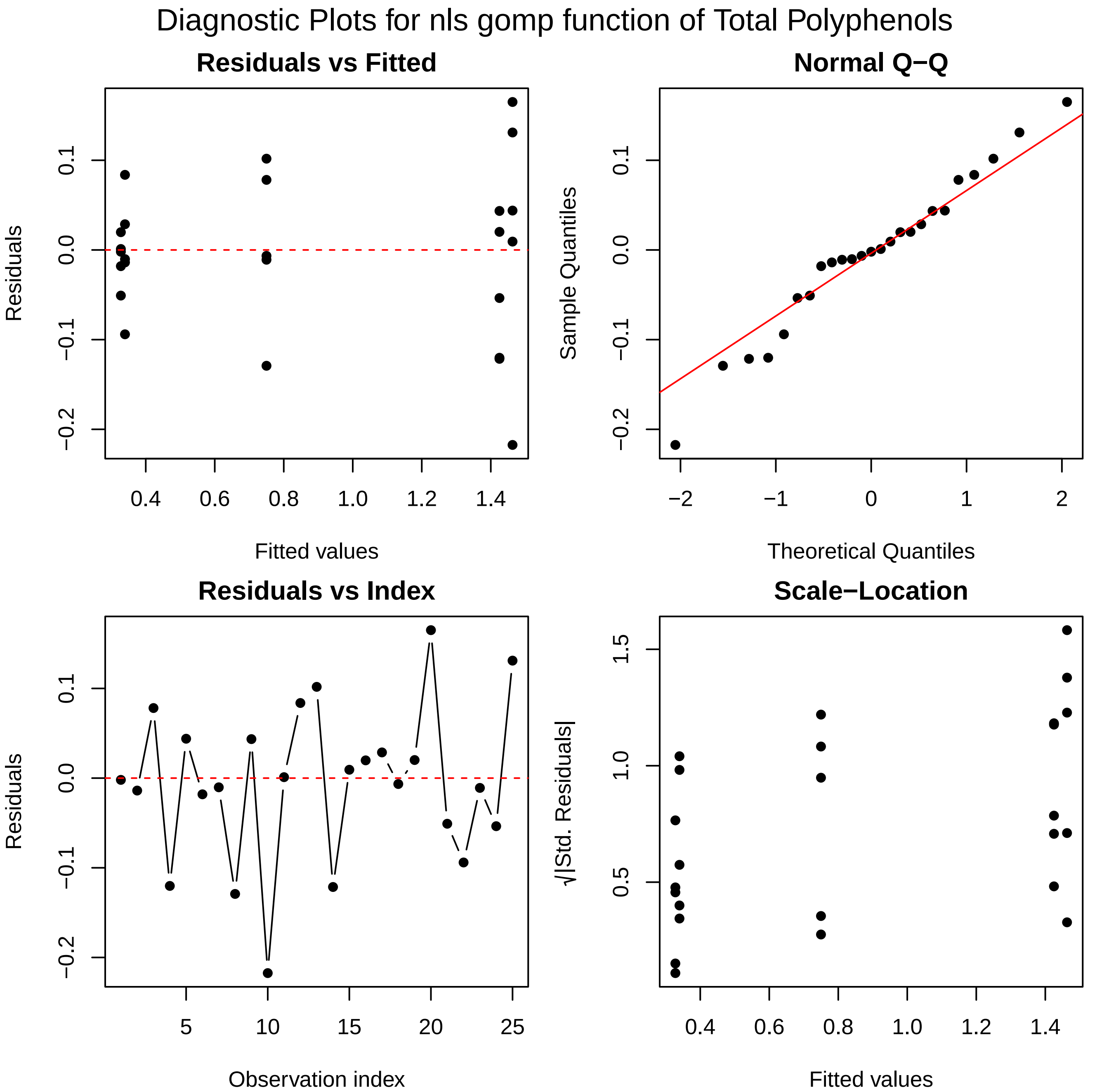
Diagnostic Plots for nls gomp function of Total Polyphenols
Residuals vs Fitted
Residuals
Fitted values
Normal Q−Q
Sample Quantiles
Theoretical Quantiles
Residuals vs Index
Residuals
Observation index
Scale−Location
√|Std. Residuals|
Fitted values

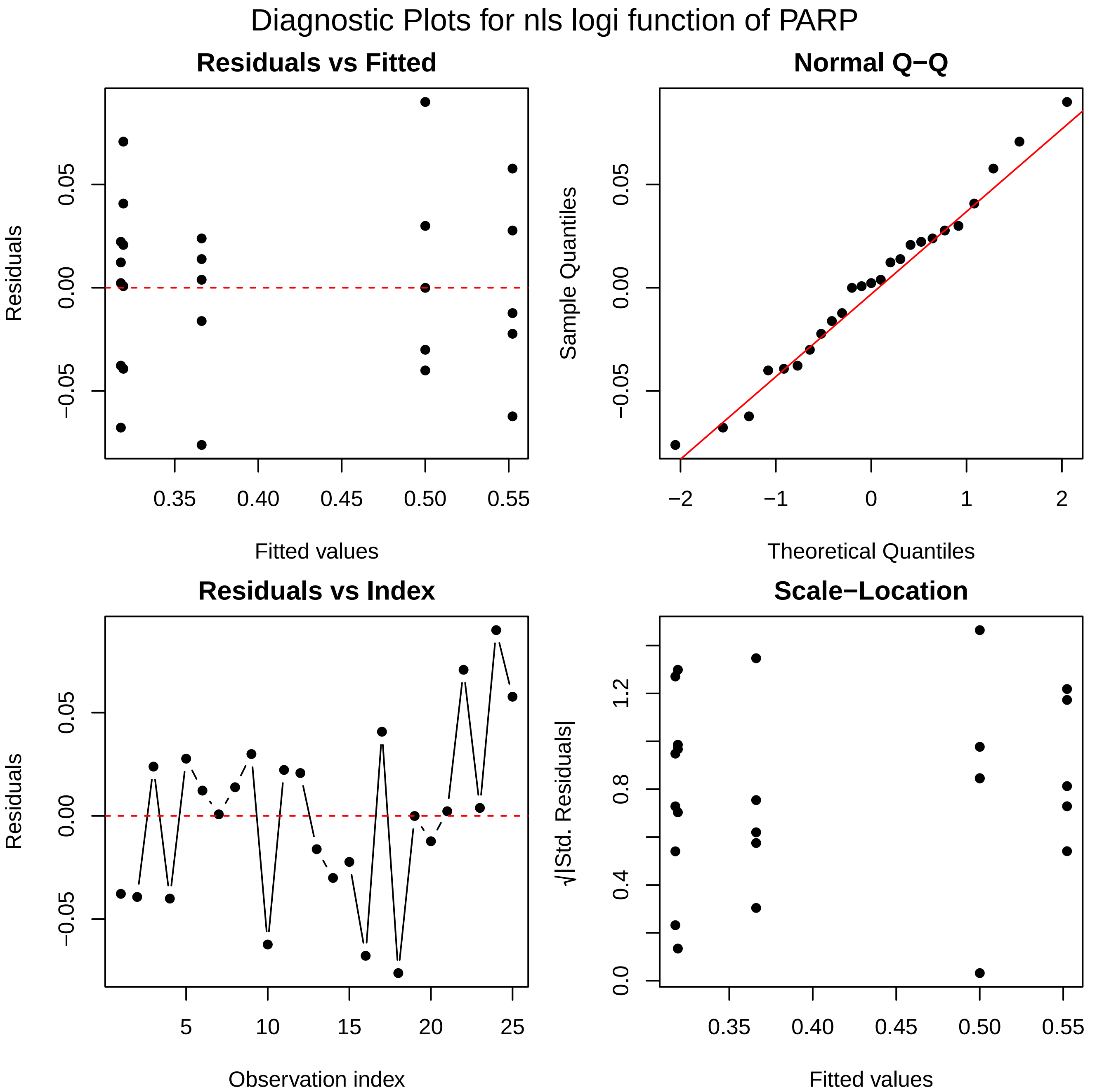

Diagnostic Plots for nls logi function of PARP
Residuals vs Fitted
Residuals
Fitted values
0.35
0.40
0.45
0.50
0.55
0.05
0.00
−0.05
Normal Q−Q
Sample Quantiles
Theoretical Quantiles
−2
−1
0
1
2
Residuals vs Index
Observation index
5
10
15
20
25
Scale−Location
√|Std. Residuals|
0.0
0.4
0.8
1.2
