# Supplementary material for: Antioxidant Defenses and Poly(ADP-Ribose) Polymerase (PARP) Activity Provide “Radioresilience” Against Ionizing Radiation-Induced Stress in Dwarf Bean Plants
Source: Antioxidants (Basel). 2025 Feb 25;14(3):261. doi: 10.3390/antiox14030261 (PMC11939814; doi:10.3390/antiox14030261)
Supplement: Supplementary file 1 [file antioxidants-14-00261-s001.zip › Figure_S5.pdf]

Diagnostic Plots for nls gomp function of fv/fm

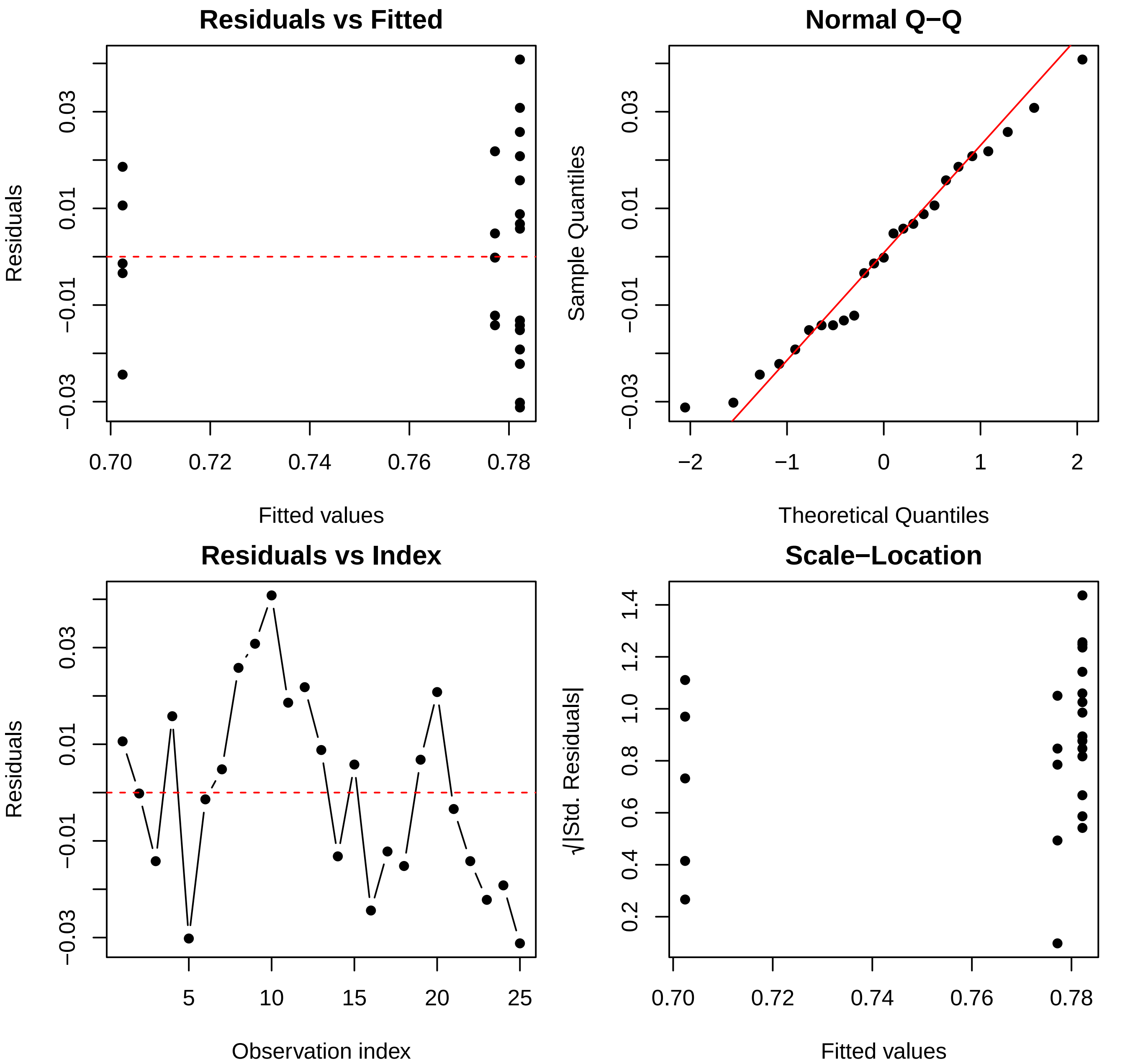

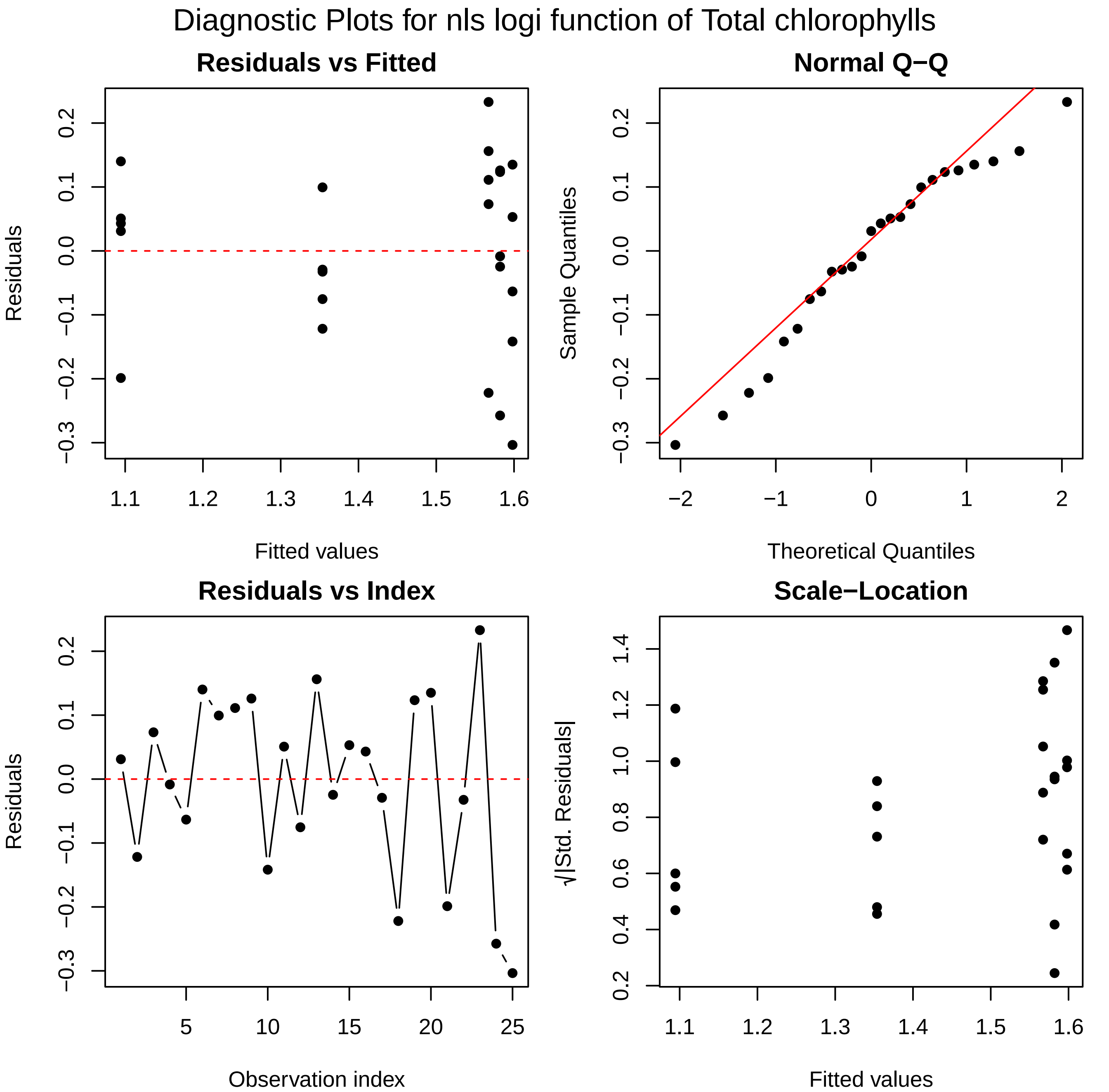
Diagnostic Plots for nls logi function of Total chlorophylls
Residuals vs Fitted
Residuals
Fitted values
Normal Q−Q
Sample Quantiles
Theoretical Quantiles
Residuals vs Index
Residuals
Observation index
Scale−Location
√|Std. Residuals|
Fitted values

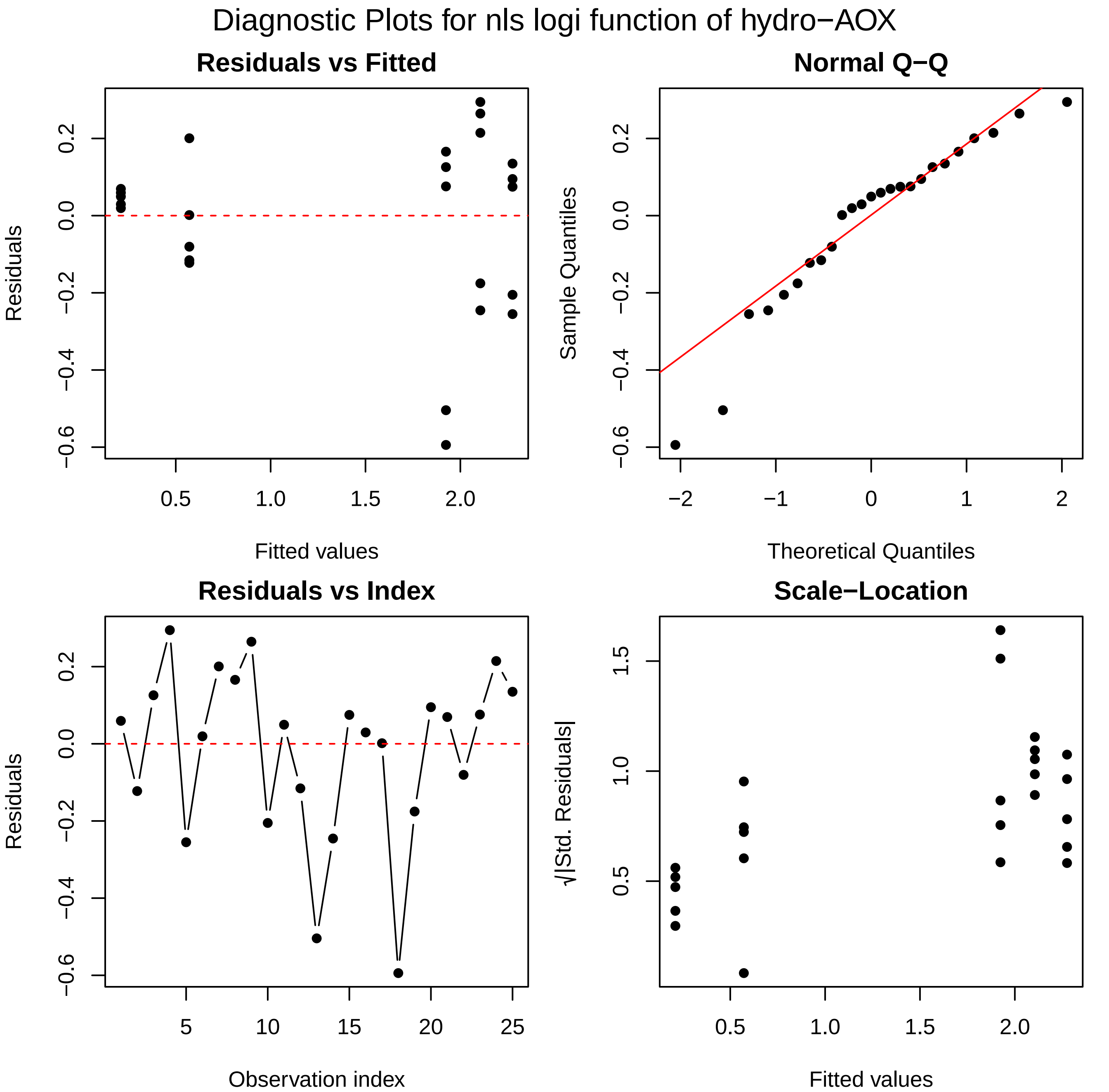

Diagnostic Plots for nls logi function of hydro−AOX
Residuals vs Fitted
Residuals
Fitted values
0.2
0.0
−0.2
−0.4
−0.6
0.5
1.0
1.5
2.0
Normal Q−Q
Sample Quantiles
Theoretical Quantiles
−2
−1
0
1
2
Residuals vs Index
Observation index
5
10
15
20
25
Scale−Location
√|Std. Residuals|
0.5
1.0
1.5

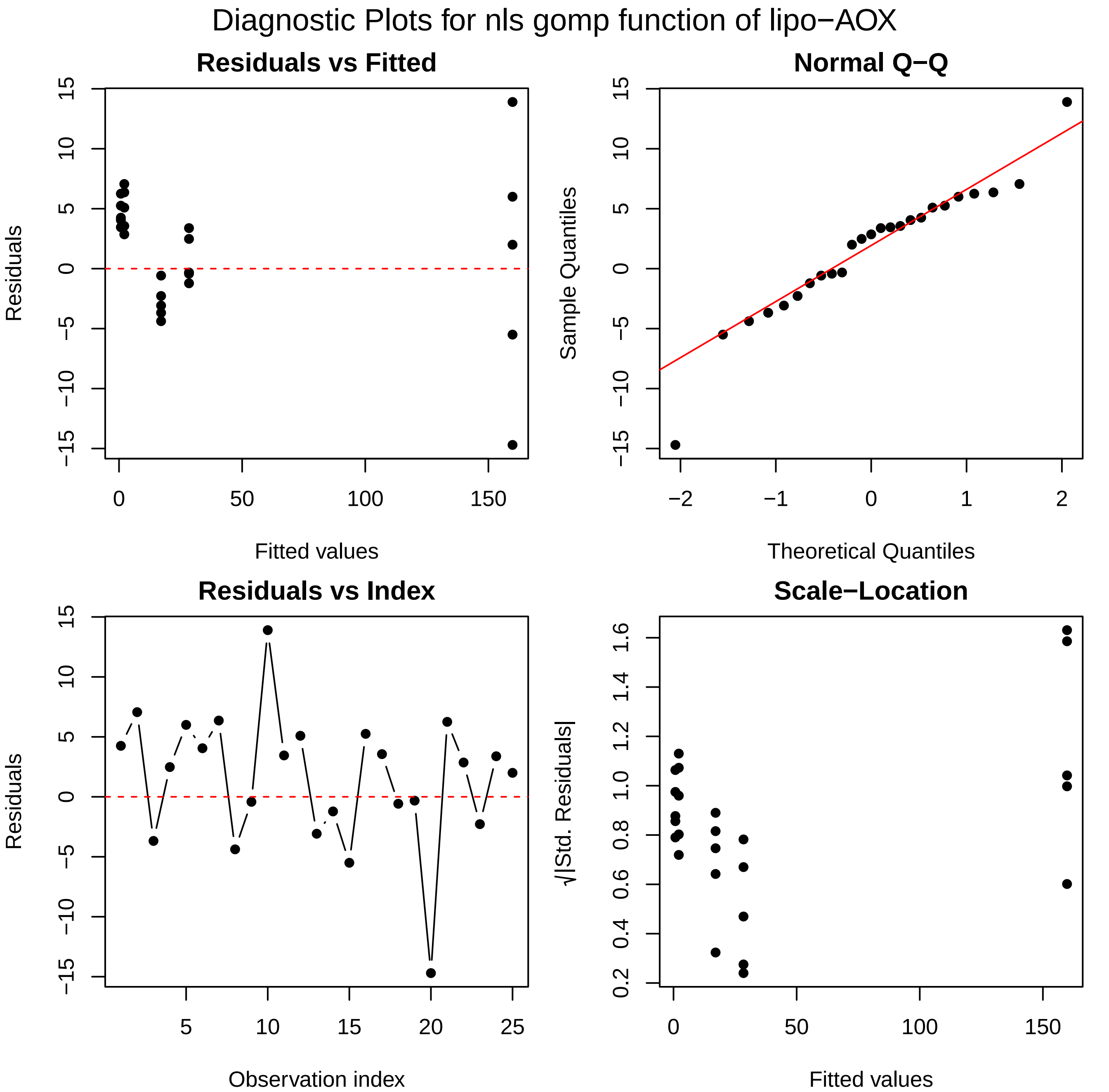

Diagnostic Plots for nls gomp function of lipo−AOX
Residuals vs Fitted
Residuals
Fitted values
15
10
5
0
−5
−10
−15
0
50
100
150
Normal Q−Q
Sample Quantiles
Theoretical Quantiles
−2
−1
0
1
2
Residuals vs Index
Residuals
Observation index
5
10
15
20
25
Scale−Location
√|Std. Residuals|
Fitted values
0.2
0.4
0.6
0.8
1.0
1.2
1.4
1.6

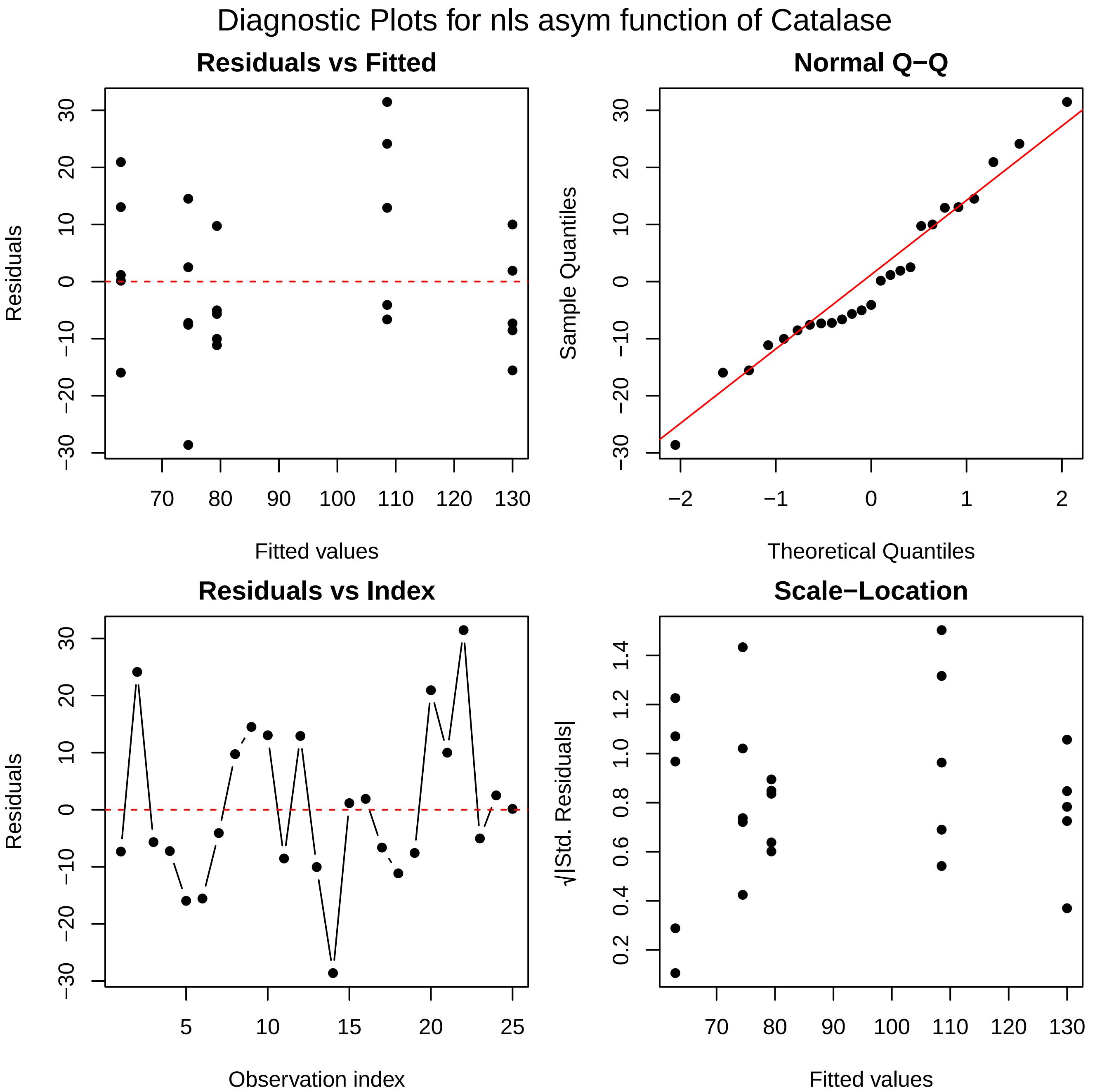
Diagnostic Plots for nls asym function of Catalase
Residuals vs Fitted
Residuals
Fitted values
Normal Q−Q
Sample Quantiles
Theoretical Quantiles
Residuals vs Index
Residuals
Observation index
Scale−Location
√|Std. Residuals|
Fitted values

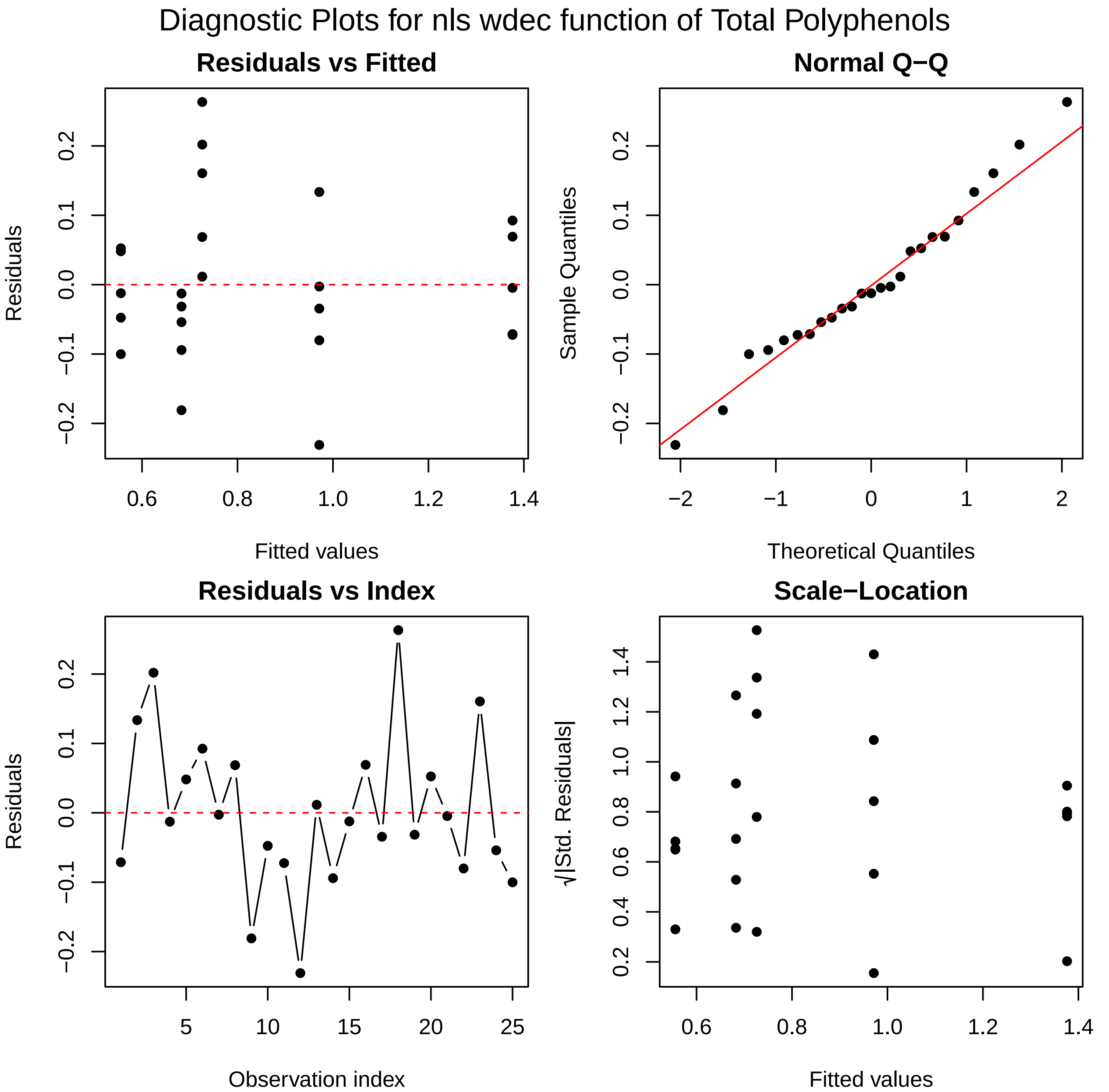
Diagnostic Plots for nls wdec function of Total Polyphenols
Residuals vs Fitted
Residuals
Fitted values
−0.2
−0.1
0.0
0.1
0.2
0.6
0.8
1.0
1.2
1.4
Normal Q−Q
Sample Quantiles
Theoretical Quantiles
−2
−1
0
1
2
Residuals vs Index
Observation index
5
10
15
20
25
Scale−Location
√|Std. Residuals|
0.2
0.4
0.6
0.8
1.0
1.2
1.4

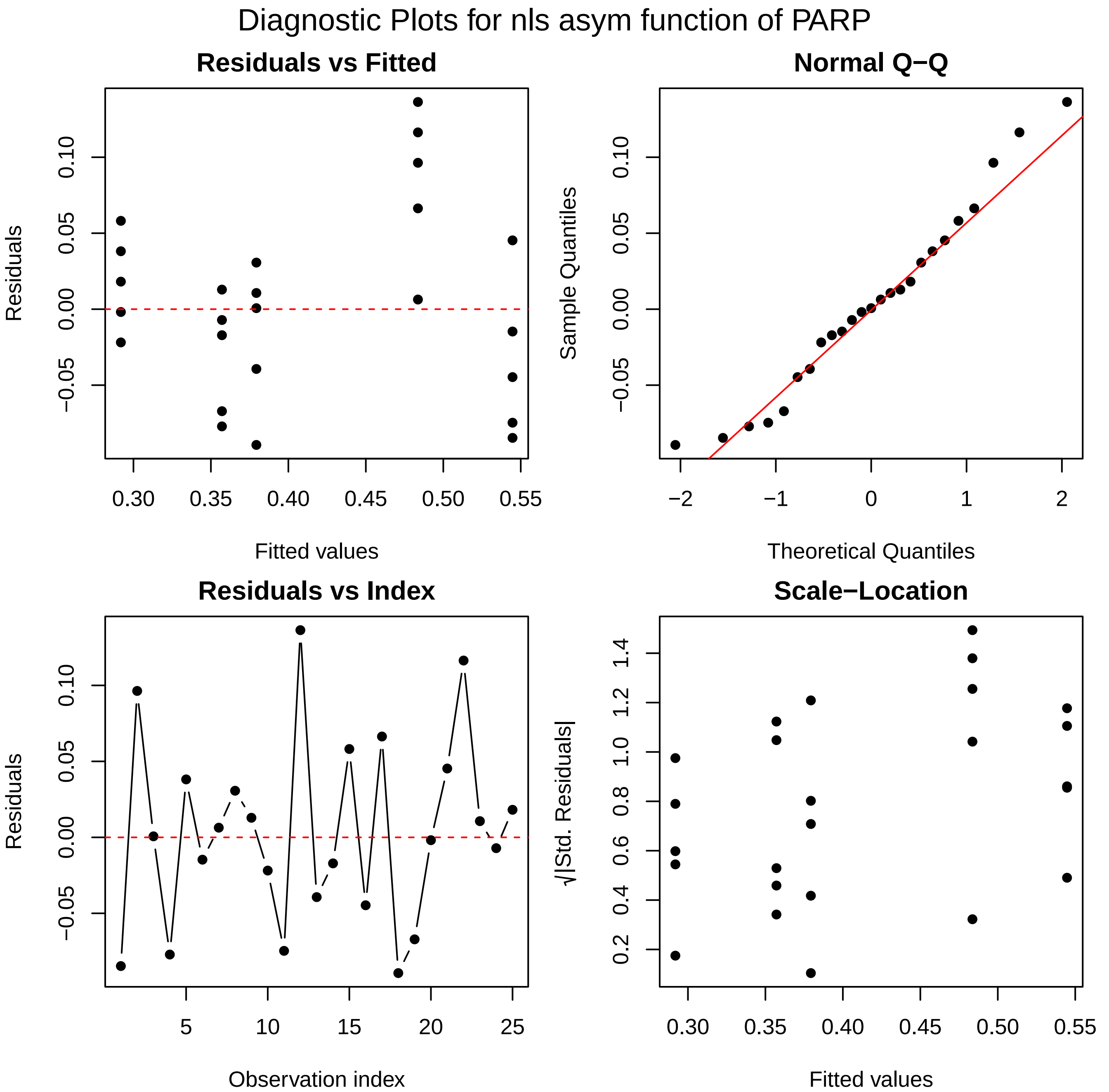
Diagnostic Plots for nls asym function of PARP
Residuals vs Fitted
Residuals
Fitted values
Normal Q−Q
Sample Quantiles
Theoretical Quantiles
Residuals vs Index
Residuals
Observation index
Scale−Location
√|Std. Residuals|
Fitted values
