# Supplementary material for: Antioxidant Defenses and Poly(ADP-Ribose) Polymerase (PARP) Activity Provide “Radioresilience” Against Ionizing Radiation-Induced Stress in Dwarf Bean Plants
Source: Antioxidants (Basel). 2025 Feb 25;14(3):261. doi: 10.3390/antiox14030261 (PMC11939814; doi:10.3390/antiox14030261)
Supplement: Supplementary file 1 [file antioxidants-14-00261-s001.zip › Supplementary tables.pdf]

Table S1. Master equation table. **Functional form (n parameters):** generic functional form and number of parameters to fit; **Rationale for choice:** why the function has been chosen **Function:** function name as referred in the text **Equation:** equation of the corresponding function.

Functional form (n. of parameters)	Rationale for choice	Function	Equation
Simple (2)	Prevent over-explanation	Linear	$a + b \cdot x$
Simple (2)	Prevent over-explanation	Exponential decay	$a \cdot e^{-b \cdot x}$
Simple (2)	Prevent over-explanation	Power law	$a \cdot x^{b}$
Asymptotic (3)	Test the hypothesis of saturation dynamics	Asymptotic exponential decay	$a + (y_0 - a) \cdot e^{-k \cdot x}$
Asymptotic (3)	Test the hypothesis of saturation dynamics	Weibull decay	$a \cdot e^{-(b \cdot x)^{e}}$
Logistic (3)	Evaluate the presence of a dose threshold	Logistic	$\frac{l}{1 + e^{-k \cdot (x - x_0)}}$
Logistic (3)	Evaluate the presence of a dose threshold	Gompertz	$a \cdot e^{-b \cdot e^{-c \cdot x}}$

Table S2. Pairwise hypothesis testing for differences between control values and recovery time points across variables. **Variable**: variable name; **dai**: day after irradiation (recovery time point); **p.value**: raw p value; **adj.p**: adjusted p value; **ci.low**, **ci.high**: extremes of the 95% confidence interval; **estimate**: delta of medians; **method**: applied method.

Variable	dai	p.value	adj.p	ci.low	estimate	ci.high	method
F_v/F_m	0	8E-03	**0.04**	-0.13	-0.10	-0.07	Wilcoxon rank sum exact test
F_v/F_m	*3*	7E-02	0.30	-0.05	-0.02	0.00	Wilcoxon rank sum test w/ continuity correction
F_v/F_m	*10*	2E-01	0.45	-0.06	-0.02	0.01	Wilcoxon rank sum exact test
F_v/F_m	*12*	4E-01	0.84	-0.05	-0.01	0.02	Wilcoxon rank sum exact test
F_v/F_m	*20*	5E-01	0.84	-0.06	-0.02	0.03	Wilcoxon rank sum exact test
Total chlorophylls	0	8E-03	**0.04**	-0.62	-0.38	-0.16	Wilcoxon rank sum exact test
Total chlorophylls	*3*	1E-01	0.38	-0.42	-0.18	0.02	Wilcoxon rank sum exact test
Total chlorophylls	*10*	3E-01	0.93	-0.16	0.17	0.38	Wilcoxon rank sum exact test
Total chlorophylls	*12*	4E-01	0.93	-0.19	0.07	0.28	Wilcoxon rank sum exact test
Total chlorophylls	*20*	8E-01	0.93	-0.24	0.01	0.29	Wilcoxon rank sum exact test
hydro-AOX	0	8E-03	**0.04**	-3.14	-2.96	-2.44	Wilcoxon rank sum exact test
hydro-AOX	3	8E-03	**0.04**	-2.91	-2.71	-2.21	Wilcoxon rank sum exact test
hydro-AOX	10	8E-03	**0.04**	-1.97	-1.30	-0.70	Wilcoxon rank sum exact test
hydro-AOX	12	8E-03	**0.04**	-1.44	-0.93	-0.38	Wilcoxon rank sum exact test
hydro-AOX	20	8E-03	**0.04**	-1.28	-0.93	-0.35	Wilcoxon rank sum exact test
lipo-AOX	0	8E-03	**0.04**	-215.00	-203.00	-175.00	Wilcoxon rank sum exact test
lipo-AOX	3	8E-03	**0.04**	-212.77	-200.80	-172.77	Wilcoxon rank sum exact test

lipo-AOX	10	8E-03	**0.04**	-206.00	-194.20	-166.00	Wilcoxon rank sum exact test
lipo-AOX	12	8E-03	**0.04**	-191.90	-178.20	-151.90	Wilcoxon rank sum exact test
lipo-AOX	20	8E-03	**0.04**	-64.90	-44.20	-16.30	Wilcoxon rank sum exact test
PARP	0	8E-03	**0.04**	0.13	0.20	0.28	Wilcoxon rank sum exact test
PARP	3	8E-03	**0.04**	0.17	0.27	0.34	Wilcoxon rank sum exact test
PARP	*10*	7E-02	0.22	-0.03	0.06	0.13	Wilcoxon rank sum test with continuity correction
PARP	*12*	3E-01	0.59	-0.05	0.02	0.09	Wilcoxon rank sum test with continuity correction
PARP	*20*	9E-01	0.92	-0.05	0.01	0.07	Wilcoxon rank sum test with continuity correction
Catalase	0	8E-03	**0.04**	68.95	79.32	96.15	Wilcoxon rank sum exact test
Catalase	3	8E-03	**0.04**	55.44	77.62	96.15	Wilcoxon rank sum exact test
Catalase	10	8E-03	**0.04**	21.21	27.89	45.29	Wilcoxon rank sum exact test
Catalase	12	3E-02	**0.04**	2.03	23.89	45.15	Wilcoxon rank sum exact test
Catalase	20	2E-02	**0.04**	3.15	20.76	40.04	Wilcoxon rank sum exact test
Total Polyphenols	0	8E-03	**0.04**	0.97	1.05	1.16	Wilcoxon rank sum exact test
Total Polyphenols	3	8E-03	**0.04**	0.41	0.61	0.78	Wilcoxon rank sum exact test
Total Polyphenols	10	8E-03	**0.04**	0.41	0.56	0.66	Wilcoxon rank sum exact test
Total Polyphenols	12	8E-03	**0.04**	0.18	0.30	0.36	Wilcoxon rank sum exact test
Total Polyphenols	20	8E-03	**0.04**	0.13	0.23	0.30	Wilcoxon rank sum exact test

Table S3. Fitted parameters value for best dose/response dynamics curve fittings. **Variable** name of the variable, **Function** function name, **Fit type** type of fit, **x1** first parameter, **x2** second parameter, **x3** third parameter
A.exp.d: Asymptotic exponential decay; Exp: exponential; Weibull.d: Weibull decay.

Variable	Function	Fit type	x1	x2	x3
F_v/F_m	Linear	robust	a = 0.8	b = -0.002	/
Total chlorophylls	Exp. decay	robust	a = 1.58	b = 0.01	/
hydro-AOX	A.exp.d	robust	a = 0.25	y0 = 2.67	k = 0.12
lipo-AOX	Weibull.d	robust	a = 202.24	b = 0.65	c = 0.29
Catalase	Gompertz	standard	a = 133.45	b = 1.11	c = 0.06
Total Polyphenols	Gompertz	robust	a = 1.46	b = 1.50	c = 0.08
PARP	Logistic	robust	l = 0.56	k = 0.04	x0 = -7.08

Table S4. Fitted parameters value for best recovery dynamics curve fittings. **Variable** name of the variable, **Function** function name, **Fit type** type of fit, **x1** first parameter, **x2** second parameter, **x3** third parameter.

A.exp.d: Asymptotic exponential decay; Weibull.d: Weibull decay.

Variable	Function	Fit type	x1	x2	x3
F_v/F_m	Gompertz	robust	a = 0.78	b = 0.11	c = 0.94
Total chlorophylls	Logistic	robust	l = 1.60	k = 0.31	x0 = -2.49
hydro-AOX	Logistic	robust	l = 2.28	k = 0.40	x0 = 5.77
lipo-AOX	Gompertz	standard	a = 43398.46	b = 10.97	c = 0.03
Catalase	A.exp.d	robust	a = 55.04	y0 = 129.99	k = 0.11
Total Polyphenols	Weibull.d	robust	a - 1.38	b = 0.04	c = 0.50
PARP	Gompertz	robust	a = 0.19	y0 = 0.54	k = 0.06
