# Supplementary figures and images for: Antioxidant Defenses and Poly(ADP-Ribose) Polymerase (PARP) Activity Provide “Radioresilience” Against Ionizing Radiation-Induced Stress in Dwarf Bean Plants
Source: Antioxidants (Basel). 2025 Feb 25;14(3):261. doi: 10.3390/antiox14030261 (PMC11939814; doi:10.3390/antiox14030261)

# Dose/response dataset

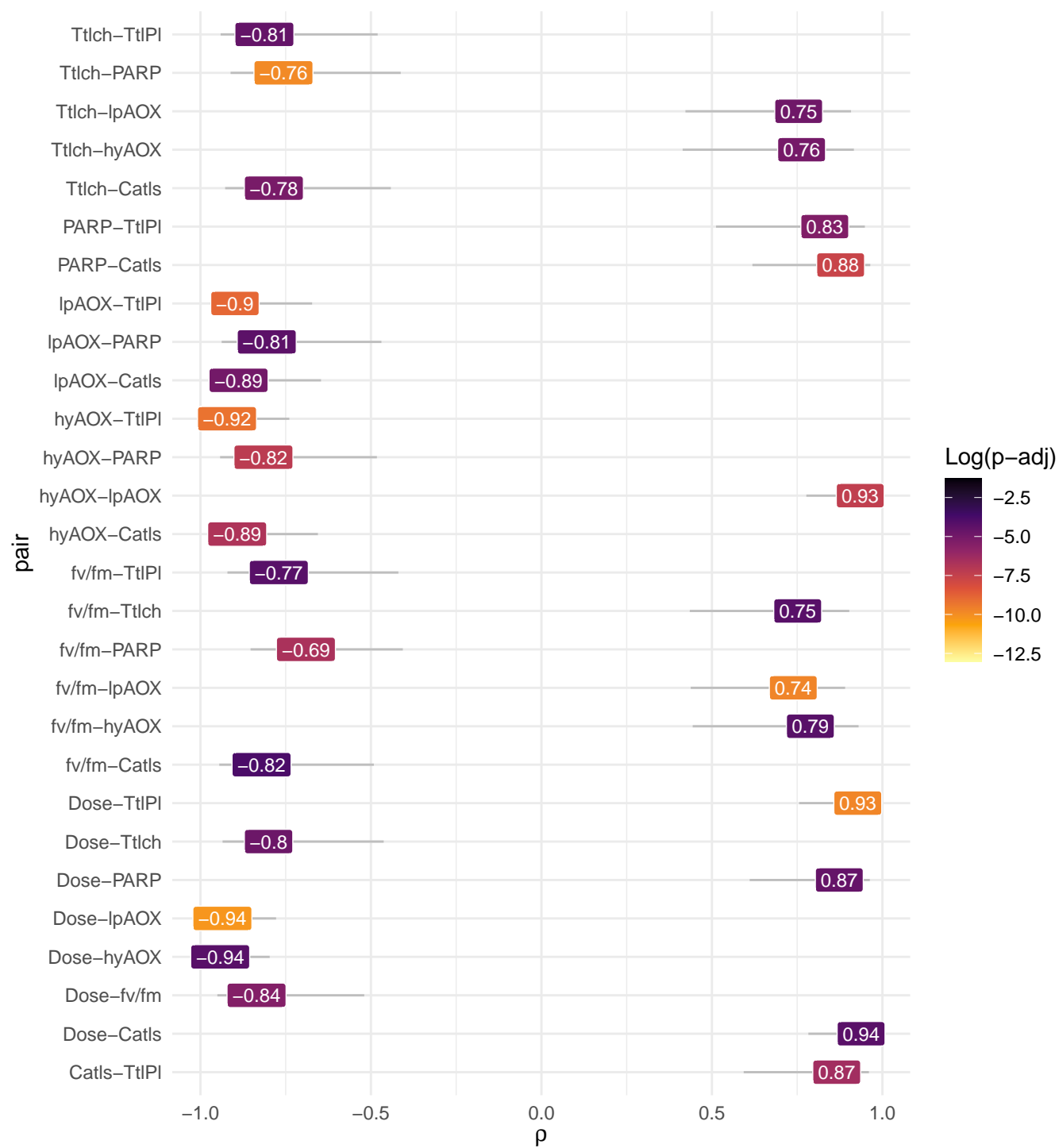

Supplement: Supplementary file 1 [file antioxidants-14-00261-s001.zip › Figure_S3.pdf]

# Recovery dataset

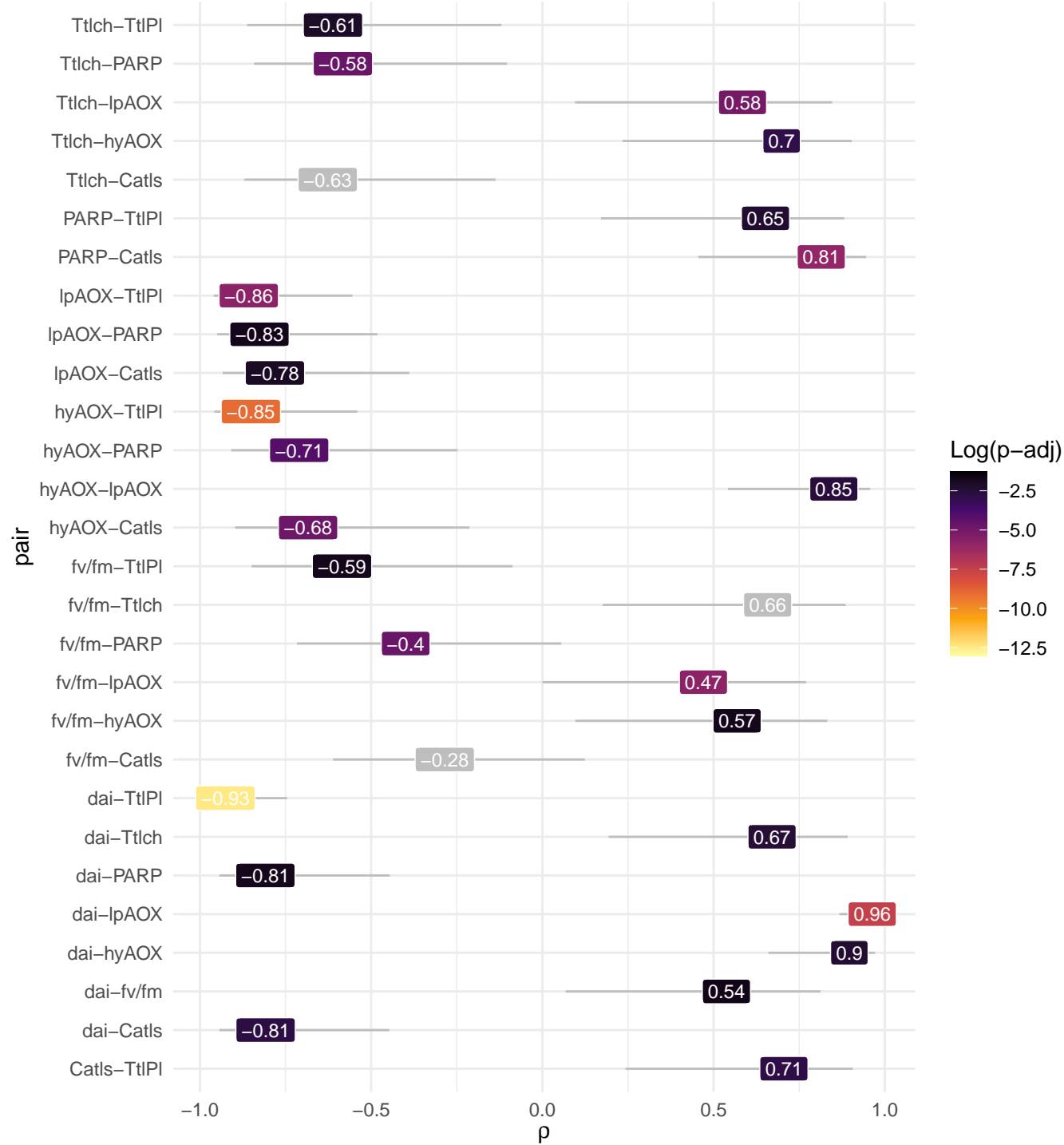

Supplement: Supplementary file 1 [file antioxidants-14-00261-s001.zip › Figure_S4.pdf]
